# Identifying and Analyzing Bot-Generated Responses in Online Health Care Surveys: Methodological Study

**DOI:** 10.2196/73622

**Published:** 2026-03-05

**Authors:** Emily Hamovitch, Kaileah McKellar, Walter P Wodchis

**Affiliations:** 1 Institute of Health Policy, Management and Evaluation, University of Toronto, 155 College Street, Toronto, ON, M5T 3M6, Canada, 1 (416) 978-4326; 2 Institute for Better Health, Trillium Health Partners, Mississauga, ON, Canada

**Keywords:** bots, online surveys, data integrity, patient-reported outcome measures, patient-reported experience measures, health care research, digital health, survey validity

## Abstract

**Background:**

The increasing reliance on online surveys for collecting patient-reported feedback for health care research has led to growing concerns over fraudulent responses generated by bots. These automated responses threaten data integrity by fabricating survey results, distorting statistical analyses, and potentially misguiding policy decisions. Addressing this issue is critical for maintaining the validity of research findings that inform health care practice and policy.

**Objective:**

This study aimed to develop a robust set of criteria for identifying bot-generated responses in online health care surveys and to examine how these responses impact data quality. We then explored differences in survey results between probable human and suspected bot respondents in a survey assessing patient-reported outcome measures and patient-reported experience measures within a geographic region in Ontario, Canada.

**Methods:**

A survey was conducted from July to October 2023 using Research Electronic Data Capture (REDCap; Vanderbilt University), distributed with a generic link via email, and later shared on social media. The survey collected data on health care use, patient experiences, health outcomes, digital health care engagement, and demographics. A 3-tier classification system was developed to detect bot responses based on predefined “red flags,” including duplicate open-ended responses, inconsistent demographic reporting, identical timestamps, and location discrepancies. Quantitative analysis included chi-square tests to assess differences between probable human and suspected bot responses and Spearman correlation tests to examine relationships among health care indicators.

**Results:**

Analysis included 1154 responses, with 58% (n=668) classified as suspected bot-generated. The most frequent suspected bot-identification criterion was duplicated open-ended responses (293/668, 44%). Chi-square tests revealed statistically significant differences (*P*<.05) between suspected bots and probable humans across most survey items. Suspected bots demonstrated response patterns concentrated in the middle of Likert scales, whereas probable humans were more likely to select extreme values. Correlation analyses showed that expected relationships between key health indicators (eg, depression symptoms) were present in probable human responses but reversed in suspected bot-generated data, highlighting the potential for compromised validity in unfiltered survey datasets.

**Conclusions:**

The findings underscore the necessity of implementing bot prevention and detection methods in online health care surveys to preserve data integrity. Failure to do so risks distorting research conclusions, particularly in health equity studies where demographic misclassification may bias results. The study highlights effective bot detection strategies, including open-text analysis, timestamp evaluation, and geographic validation, and recommends integrating these techniques into survey design. As bots continue to evolve, ongoing advancements in bot prevention and detection will be crucial to maintaining the reliability of digital health research.

## Introduction

Patient-reported outcome measures (PROMs) and patient-reported experience measures (PREMs) are invaluable tools for understanding and improving health care from the patient’s perspective. PROMs assess health status, functional abilities, symptoms, and quality of life directly from patients, often through self-reported questionnaires, providing insights into the impact of interventions and overall care quality [1-3]. PREMs, on the other hand, capture patients’ perceptions of their health care experience, focusing on whether key processes occurred and how care was delivered [2]. These measures are essential for implementing person-centered care, as they enable health care providers to align their practices with patient needs and experiences [4]. PROMs and PREMs have also been incorporated into care routines to support quality improvement initiatives [4]. By prioritizing patient-reported data, health care systems can address inequities, promote patient-centered and value-based care, and ensure that care delivery aligns with the priorities and needs of diverse patient populations [4].

A common approach to collecting PROMs and PREMs is through online surveys. Online surveys have become an increasingly prominent tool for research, particularly in social and behavioral health fields, due to their efficiency, cost-effectiveness, and ability to overcome challenges faced by traditional data collection methods such as face-to-face, mail, or telephone surveys [5,6]. These challenges include declining response rates, rising costs, and the need for social distancing during events like the COVID-19 pandemic [5,7-10]. Internet-based platforms such as Qualtrics (Qualtrics International) Inc., SurveyMonkey (Momentive Inc.), and Research Electronic Data Capture (REDCap; Vanderbilt University) have enabled researchers to collect data remotely, making it possible to reach large general populations [5,7,10]. These tools reduce the resources required for data collection, minimize participant burden, and offer greater anonymity, encouraging honest disclosure of personal or sensitive information [7,10]. Additionally, the limited availability of a sampling frame with accurate contact information has necessitated the use of distribution methods involving generic survey links and promotion through online platforms and social media. Unfortunately, recruiting participants through generic links creates susceptibility to data quality issues [11].

While early guidance on online surveys for health research warned of the risk of hoax responses [12], the increasing use of online surveys has been accompanied by a growing concern over fraudulent responses, particularly from bots - applications that automatically complete surveys – producing insincere data and inaccurate results [13]. These bots are capable of mimicking human behavior by selecting multiple-choice answers and generating plausible open-ended responses, making them difficult to detect. Bots can submit hundreds of fabricated responses within hours, potentially overwhelming researchers with invalid data and causing significant financial loss if participant incentives are misallocated [8]. Their presence threatens data quality, which can distort statistical analyses and compromise research findings [10]. Researchers, evaluators, and program analysts also face challenges in identifying fraudulent responses due to the increasing sophistication of bots and the anonymity of online participants [14]. Despite the implementation of data protection mechanisms to prevent bot responses, such as reCAPTCHA (Google LLC), bot programmers easily bypass these safeguards using virtual private networks or servers [7]. As bots become more prevalent and capable, addressing this issue is critical to ensuring data integrity and the validity of research findings that inform policy and practice.

This study adds to this literature by proposing a structured 3-tier classification system that integrates multiple indicators of responses by suspected bots, offering a robust and transparent protocol for identifying suspected bot responses. Additionally, this study goes beyond merely identifying suspected bot-generated responses in online surveys; it systematically examines how suspected bots influence response patterns in a health care experience survey. While existing research has focused on detecting fraudulent responses, fewer studies have examined how suspected bot-generated data may distort patient-reported experience and outcome measures in health care contexts. Our study extends this work by analyzing discrepancies between suspected bot and probable human responses in a survey of PROMs and PREMs. Such differences provide an assessment of potential bias and the relative importance of detection.

## Methods

### Survey

This study was undertaken within a broader evaluation of Ontario Health Teams (OHT), focusing on patient experiences and outcomes. A patient survey was implemented to support planning and evaluation of integrated care for attributed populations in Ontario, Canada. Consent was obtained from all respondents included in the analysis. The survey was intended for patients from a large OHT, gathering data on PREMs and PROMs. Questions included Likert scale items on health care use, health outcomes, accessing care, having someone to count on, being heard, managing health, safety, care transitions, digital health care use, demographics; as well as an open-text section to provide additional feedback. Six timestamps were programmed throughout the survey at multiple points to track survey progress. Data were collected between July 2023 and October 2023 using REDCap. REDCap is a web-based platform designed for research data capture, widely used in academic and clinical studies. The survey was distributed through the OHT’s email lists and social media platforms. Social media distribution occurred primarily through Facebook (Meta), which was the OHT’s main platform. Posts containing the survey link and QR code were shared periodically between July and October 2023, and a paid Facebook advertisement was implemented in early October to boost participation. Participants were invited to enter a draw for a $25 CAD (US $18.62) gift card as an incentive. Entry into the draw was voluntary and completed through a separate form linked on the survey’s thank-you page, allowing respondents to opt out and ensuring that contact information was stored separately from survey responses. A copy of the survey is provided in Multimedia Appendix 1.

### Suspected Bot Identification

Upon reviewing the qualitative responses and timestamp data, the research team identified clear evidence of suspected bot-generated responses in the survey. Many responses were duplicative, contained overly formal or generic language, or were nonsensical. The response patterns, combined with irregular timestamp data, confirmed the presence of suspected bots. As a result, the team developed specific criteria to systematically identify and remove suspected bot-generated responses from the dataset.

Criteria to identify suspected bots were developed using the following themes: generic and duplicative open-ended responses; concerns with location as determined by postal code (including postal codes that were invalid or nonsensical); timing irregularities; identical timestamps to other participants; illogical demographic reporting; and discrepancies in reporting health care use. In addition, a “bot takeover day” (October 10 onward) was included as a criterion, reflecting the sudden influx of suspicious responses observed on or after that date.

To identify bots in the survey data, we developed a 3-tier classification system based on specific “red flags” (concerning or questionable features or responses in the data). This approach builds on prior research that has typically relied on individual indicators or sets of criteria considered in parallel to flag fraudulent responses [5,7-9]. By combining multiple indicators into a structured, tiered framework, our system increases the robustness and transparency of suspected bot identification. Tier 1 criteria identified a respondent as a suspected bot if any single criterion occurred on its own (eg, duplicate open-ended responses that were exact full-text matches, not brief “No”-type replies, invalid postal codes, or identical timestamps). Tier 2 classified a respondent as a suspected bot if 2 or more criteria from its list were observed. For example, one tier 2 criterion included having a valid postal code outside of the region where participants received health care services. This criterion was not sufficient on its own to categorize a respondent as a suspected bot, as some participants may legitimately live elsewhere and receive care in the area where the participants were sampled. However, when combined with other criteria, it strengthened the likelihood of being a suspected bot-generated response. Finally, tier 3 flagged respondents as suspected bots if any tier 3 criterion was combined with at least 2 additional criteria from either tier 2 or tier 3, resulting in a total of 3 or more issues. One Tier 3 indicator reflected a sudden influx of suspicious responses observed on October 10, 2023 (“bot takeover day”). This indicator was not used as a standalone criterion and had minimal influence on classification: more than 99% of suspected bot responses were already identified using Tier 1 and/or Tier 2 criteria alone, with only 3 additional responses flagged through the use of this indicator. Demographic characteristics such as race or ethnicity, gender, and sexual orientation were not used as criteria for suspected bot identification. Demographic variables were analyzed only after classification to describe group-level differences between suspected bots and probable humans.

One criterion for identifying suspected bots was the presence of open-text responses that were deemed to be either (1) generic or informal, or (2) had a similar sentence structure to other responses. To evaluate this more subjective criterion, 2 reviewers (LC and SU) independently coded open-ended responses based on these criteria. Responses were categorized as suspected bots if reviewers agreed they met either criterion. When reviewers disagreed on whether open-ended responses should be classified as generic or informal, a third member of the research team (KM) provided the final determination. Interrater reliability between the 2 independent coders was substantial (Cohen κ=0.63, *P*<.001).

Although this study applied the identification criteria for a survey implemented on the REDCap platform, the 3-tier system is designed to be adaptable across different survey platforms, including Qualtrics, SurveyMonkey, and other online tools. The criteria used (such as duplicated open-text responses, timestamp irregularities, and geographic inconsistencies) are not platform-specific.

### Suspected Bot Response Analysis

To examine differences in survey responses between probable humans and suspected bots, bar charts were created to visually compare response patterns. Chi-square tests were conducted to identify significant differences in the proportions of responses between the 2 groups. For select-all-that-apply items, including “Who did you receive care from?,” responses were summarized into a mutually exclusive categorical variable prior to analysis. Respondents selecting more than 1 option were classified as having received care from “multiple providers,” while those selecting a single option were classified according to that provider type.

To account for multiple comparisons across survey items, a Bonferroni correction was applied (adjusted *α*=.05/59=0.00085) to control for Type I error. We quantified the magnitude of group differences by calculating effect sizes for each chi-square test. Cramer V was computed for all categorical comparisons, along with 95% CIs.

Additionally, Spearman correlation tests were conducted to evaluate significant relationships between 2 health indicators, analyzed separately for probable humans and suspected bots, to identify patterns that align with or deviate from expected human perception and behavior. Two sets of correlations were examined: (1) the correlation between “little interest or pleasure in doing things” and “feeling down, depressed, or hopeless,” and (2) the correlation between self-rated health and the frequency of interaction with the health system. The first pair represents the 2 core items of the Patient Health Questionnaire–2 (PHQ-2), a validated depression screener in which these items are strongly and positively correlated with one another [15]. The second pair was selected because extensive literature has shown that poorer self-rated health is associated with increased health care use, including higher rates of hospitalization and outpatient visits [16,17]. These 2 pairs, therefore, provide theoretically justified validity checks.

### Ethical Considerations

This study received approval from the University of Toronto Research Ethics Board (#38072). All procedures were conducted in accordance with institutional ethical standards and the principles of the Declaration of Helsinki. Participants provided informed consent electronically prior to beginning the survey. The consent form outlined the purpose of the study, the voluntary nature of participation, potential risks and benefits, and data management procedures. Participants were informed that they could decline to answer any question or withdraw at any time prior to survey submission. To protect privacy and confidentiality, survey responses were collected anonymously. Any contact information provided for entry into the incentive draw was submitted through a separate form and stored separately from survey data. Data were stored on secure, password-protected institutional servers accessible only to authorized members of the research team.

## Results

### Frequency of Suspected Bot Identification

Table 1 illustrates the final criteria for identifying suspected bots and the frequency with which each criterion was met among respondents ultimately classified as suspected bots or probable humans. While some probable human respondents exhibited isolated red flags, they did not meet the necessary combination of criteria required for suspected bot classification. After removing survey responses where substantive questions were not answered (n=231), 1154 survey respondents remained. Among them, 58% (n=668) were categorized as suspected bots, and 42% (n=486) were categorized as probable humans. Table 1 illustrates the number of respondents who met the eligibility criteria for each identification criterion. Overall, 529 survey respondents met the criteria for Tier 1, 570 respondents met the criteria for Tier 2 (136 of whom did not meet the criteria for Tier 1), and 551 respondents met the criteria for Tier 3 (3 of whom did not meet the criteria for Tier 1 or Tier 2).

**Table 1.** Frequency of each suspected bot identification criteria. Percentages within each tier sum to greater than 100% because a single respondent may meet multiple identification criteria.

Criteria	Assessed categories		
	Suspected bot (n)	Probable human (n)	Respondents identified as suspected bots who met criteria, n (%)
Tier 1			
Had an identical open-ended question response identical to at least one other respondent (Duplicate open-ended responses that were exact full-text matches, not brief “No”-type replies)	293	0	(293/668) 44%
Open text is generic, informal, or similar sentencing structure	49	0	(49/668) 7%
Duplicate invalid postal codes that were completed on the same day	189	0	(189/668) 28%
Duplicate out-of-province postal codes (excluding Quebec) that were completed on the same day	152	0	(152/668) 23%
Tier 2			
Identical open-ended question marked as “no” that also fell within the same day and similar time frame	80	0	(80/668) 12%
2 or more survey respondents entered their surveys within 30 seconds of one another (but not identical), and this is the case in 3 or more timestamps	340	2	(340/668) 51%
Had an identical timestamp (identical hours, minutes, and seconds) with at least one other respondent on the following timestamps:			
First timestamp	114	0	(114/668) 17%
Second timestamp	93	0	(93/668) 14%
Third timestamp	75	1	(75/668) 11%
Fourth timestamp	80	0	(80/668) 12%
Fifth timestamp	69	2	(69/668) 10%
Sixth timestamp	75	0	(75/668) 11%
Survey was completed between 1 AM and 5:00 AM Eastern Time (as identified on any of the timestamps)	154	7	(154/668) 23%
Time spent on survey is 3 minutes or less (among respondents who completed the survey)	42	11	(42/668) 6%
Invalid postal code (gibberish or numeric)	189	0	(189/668) 28%
Valid postal code but outside of Ontario	153	0	(153/668) 23%
Valid Ontario postal code but outside of the region of the primary care center in which respondents were recruited	165	5	(165/668) 25%
Discrepancy in reporting ED^a^ visits	347	55	(347/668) 52%
Between the ages of <18‐44 years but reported living in a retirement home or long-term care facility.	123	1	(123/668)18%
Tier 3			
Bot takeover day of October 10 or after	618	94	(618/668) 93%
Postal code from neighboring province	55	3	(55/668) 8%

a ED: emergency department.

In tier 1, the most commonly reported “red flag” was having an identical open-ended response to another survey respondent. In tier 2, the most common ’red flag’ was a discrepancy in reporting an emergency department visit, where respondents indicated they had received care from the emergency department in 1 question but later reported that they had not been to the emergency department in the past 12 months. There was also a high frequency of respondents completing their surveys within similar timeframes (completing 3 or more of the same sections within 30 s of another respondent).

### Differences in Responses Between Suspected Bots and Probable Humans

Overall, probable humans had a higher average percentage of missingness on all questions (mean 13% [ for probable humans vs mean 5% for suspected bots).

Chi-square tests revealed significant differences (*P*<.05) in response distributions between probable humans and suspected bots for 55 out of 59 survey questions. After Bonferroni correction for multiple comparisons (adjusted α=.00085), 50 of 59 valid comparisons remained statistically significant (Table S1 in Multimedia Appendix 2). The magnitude of effect sizes ranged from Cramer V=0.01 (“When you left the hospital were you provided with easy to follow instructions on whom to contact if you had a question about your treatment or if your condition became worse?”) to Cramer V=0.72 (“In general, do your health care providers encourage you to bring someone with you to your appointments?”), with a mean Cramer V of 0.31 (Table S1 in Multimedia Appendix 2). Among the 9 survey questions that showed no statistically significant difference between probable humans and suspected bots, most items were binary (yes or no) questions that followed branching logic and were therefore presented only to a subset of respondents. The remaining non-significant items were measured on a 5-point response scale, where probable humans tended to provide slightly less positively skewed responses (for example, being more likely to select experiencing “slight pain or discomfort” rather than “no pain or discomfort”).

**Figure 1.**
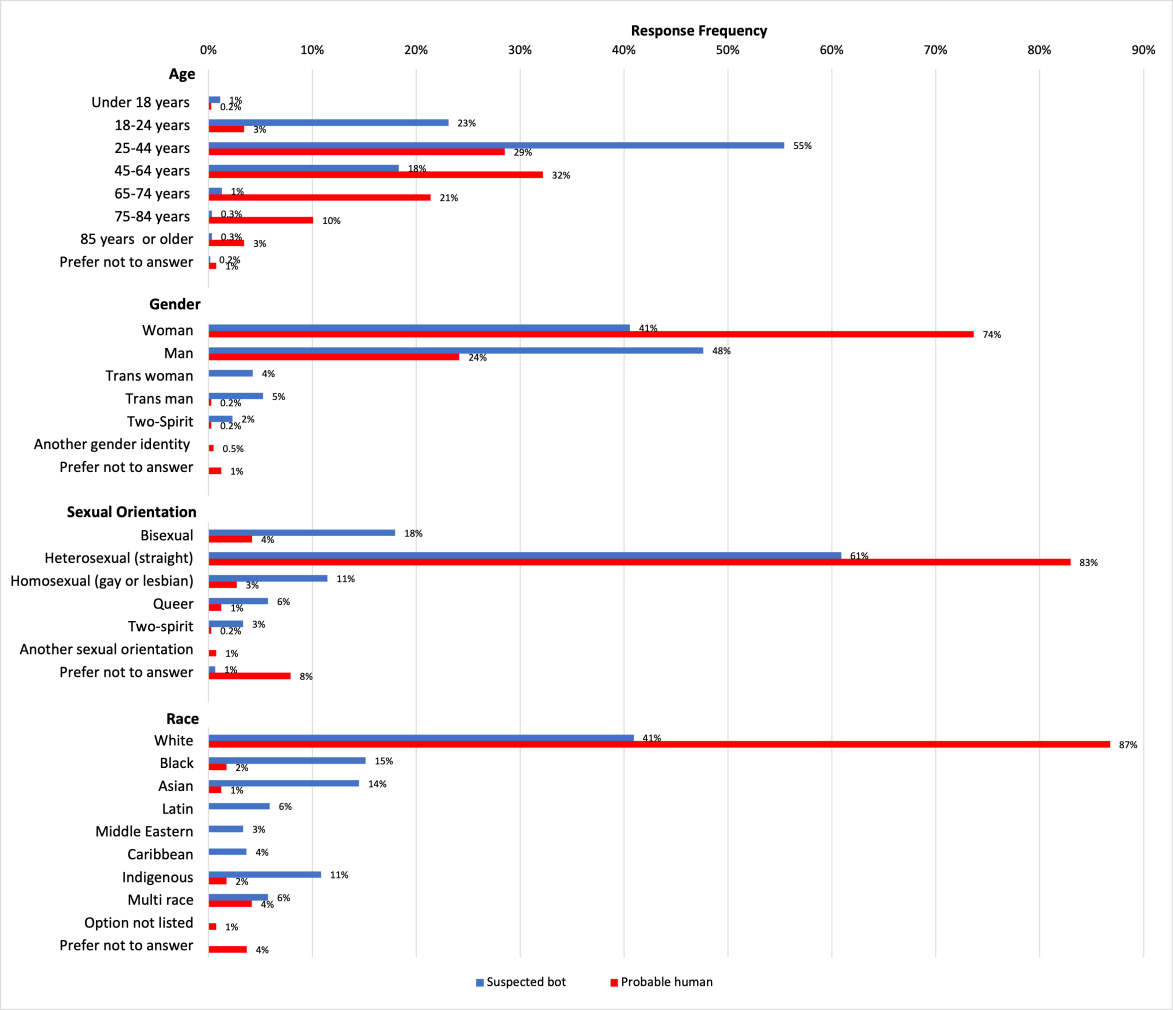
Distribution of demographics for suspected bots versus probable humans.

In terms of demographics (Figure 1), suspected bots more often reported younger ages (under 18–44 years) and being male, while probable humans more often reported being female. Both groups most frequently identified as heterosexual and White, but suspected bots more often selected minority sexual orientations (eg, bisexual, queer, and two-spirit) and racial or ethnic categories (eg, Black, Asian, Latin, and Indigenous).

**Figure 2.**
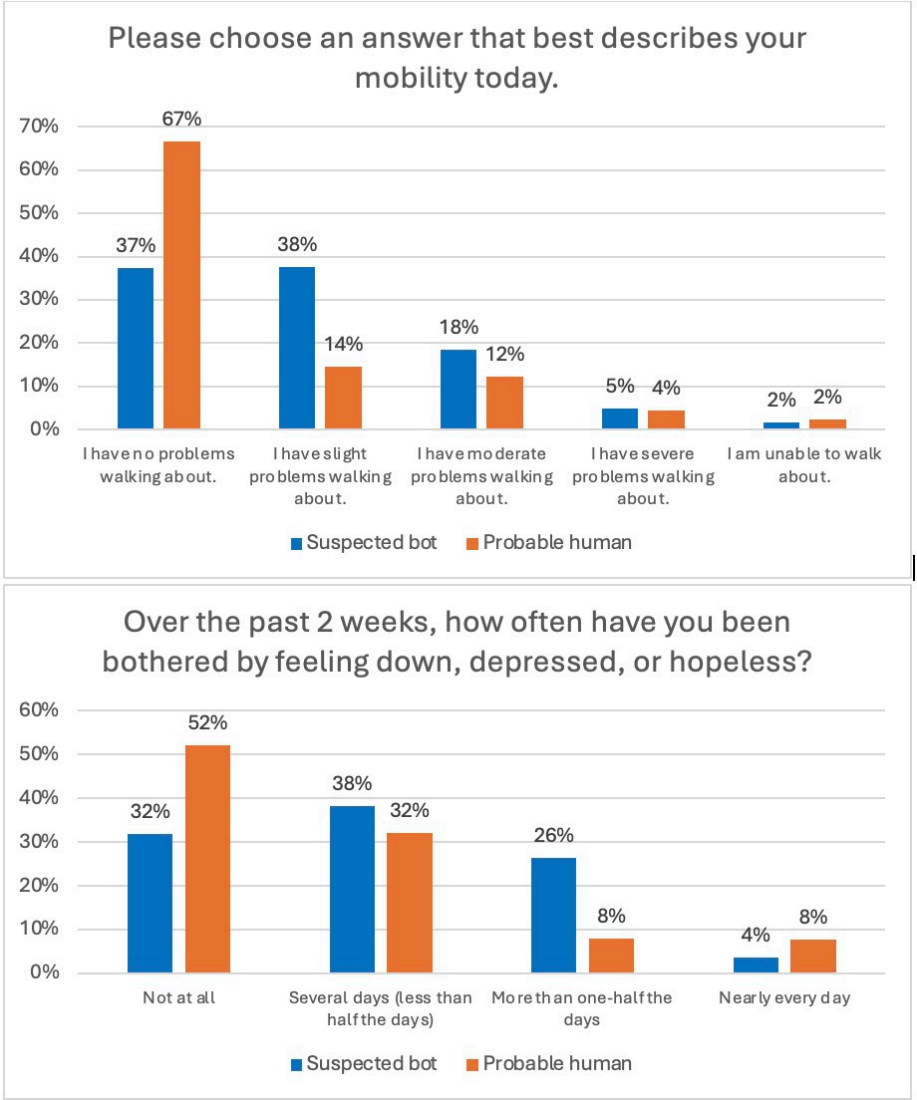
Distribution of probable human and suspected bot responses for patient-reported outcome measures.

Visualizations (Figures2  3) showed that suspected bots tended to cluster at mid-scale responses, while probable humans more often selected extreme values. For PROMs, probable humans were more likely to report the most favorable outcomes (eg, “no problems walking about”) and the least favorable (eg, “nearly every day” feeling depressed), whereas suspected bots gravitated toward midpoints (eg, “slight problems,” “several days”). Similar patterns appeared in PREMs, where suspected bots more frequently selected “somewhat” responses (eg, “somewhat easy” to access supports, “somewhat too long” to get care), while probable humans showed a greater spread across extremes.

The survey also assessed health service use, including emergency department visits, hospitalizations, and specialist care in the past year. Suspected bots more frequently reported using the emergency department and being hospitalized compared to probable humans. When asked yes or no questions about these services (such as whether an emergency department visit could have been managed by a regular provider, whether their provider was informed after hospital discharge, or whether a specialist had relevant medical information), suspected bots were more likely to respond “yes” than probable humans. This was consistent with other patterns, such as reporting “somewhat confident” when asked about managing health post-emergency department visit, while probable humans were more likely to select “very confident” or “not at all confident.”

The survey included 1 nominal question, which asked about the main reason participants had not looked at their medical records online (for those who indicated this to be the case). There did not appear to be any trend or pattern for the distribution of responses. Ironically, a substantially higher percentage of suspected bots (compared to probable humans) indicated that a reason for not having looked at medical records online was not having reliable access to the internet.

**Figure 3.**
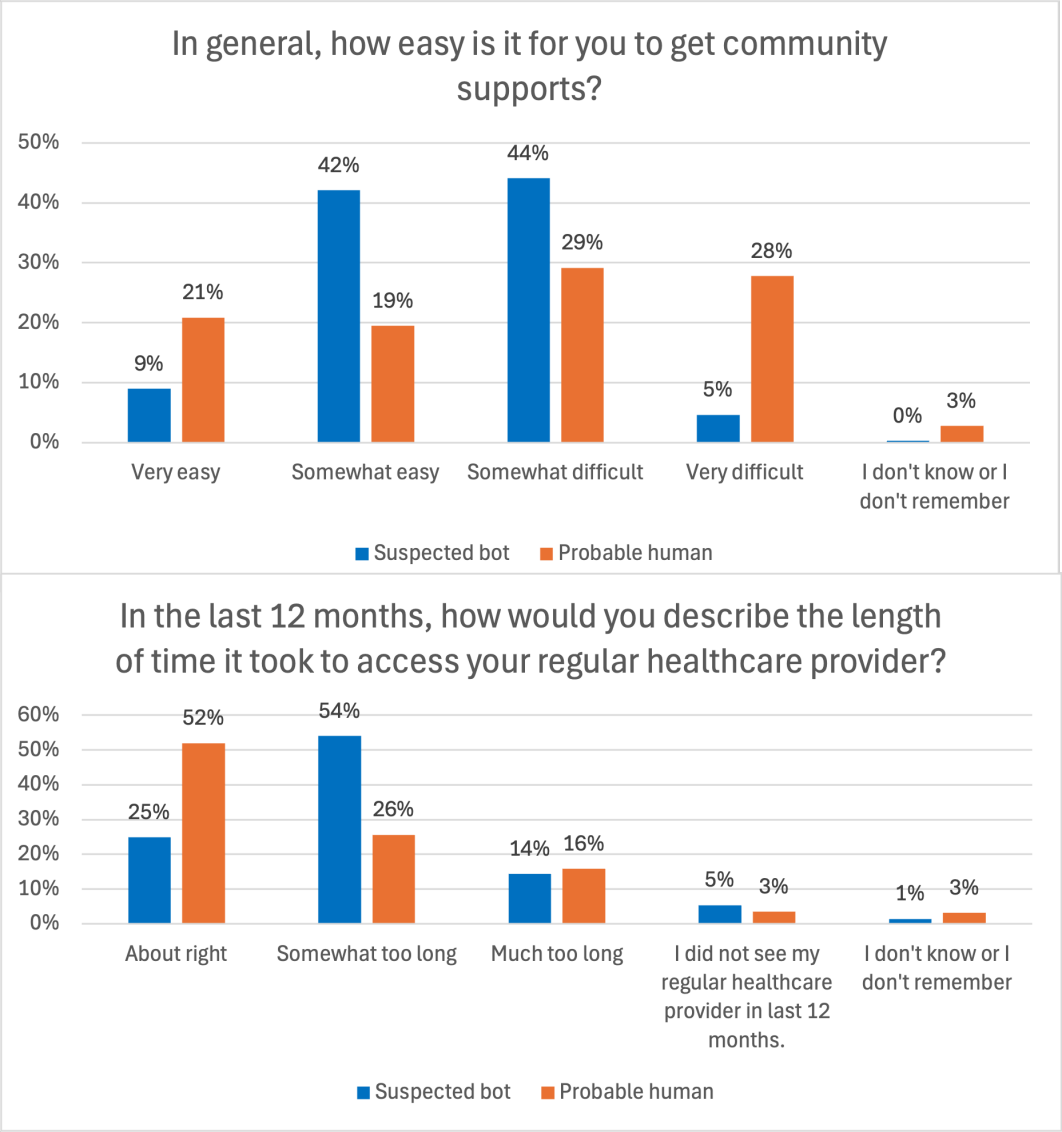
Distribution of probable human and suspected bot responses for patient-reported experience measures.

### Correlations

Spearman correlations indicated significant correlations between the indicators of having “little interest or pleasure in doing things” and “feeling down, depressed, or hopeless,” both of which are indicators of depression. For probable humans, the Spearman correlation was 0.48 (*P*<.001), indicating a moderate positive correlation between the 2 variables, whereas for suspected bots, the Spearman correlation of −0.33 (*P*<.001) indicated a moderate negative correlation between the 2 variables.

When analyzing the correlation between self-rated health (“how do you rate your own health?”) and frequency of interacting with the health system (“how often do you interact with the health system?”), a Spearman correlation of −0.07 for probable humans indicated a weak negative correlation; however, this was not significant (*P*=.15). For suspected bots, the Spearman correlation was 0.06, indicating a weak positive correlation; however, this was also not significant (*P*=.15).

## Discussion

### Principal Findings

The results of this study demonstrate the imperative need for researchers, evaluators, and program analysts in the health care field to recognize and address the potential threats posed by bots in online survey research. Failure to detect and remove bot-generated responses can lead to compromised data quality and incorrect inferences. Implementing robust data screening strategies is important for reducing fraudulent responses and improving the likelihood that valid data are retained. These responses respect the integrity of genuine participants whose experiences deserve to be accurately reported.

### Implications of Including Bot Data in Health Care Surveys

Our findings extend prior work on bot response patterns by demonstrating how these differences manifest in PROMs and PREMs, with implications for health care survey research. Across multiple survey domains—health outcomes, health care access, and experiences—probable humans were more likely than suspected bots to select the most favorable outcomes (or in some cases, the least favorable response options). In contrast, suspected bots exhibited a tendency to select responses clustered around the middle of the scale. As a result, the presence of bot-generated data could dilute the strength of human responses, skewing average scores toward the midpoint and diminishing the ability to detect meaningful trends. This issue extends beyond scaled responses; even in nominal questions, such as the reason for not accessing medical records online, suspected bots disproportionately selected specific response options, such as lacking reliable internet access. This discrepancy suggests that bot-generated data may introduce artificial patterns in categorical variables, leading to inaccurate estimates of barriers to health care access and potentially misinforming policy decisions. This distortion may undermine construct validity, as the data fail to capture real-world experiences. Additionally, probable humans demonstrated a higher level of missingness compared to suspected bots. This likely reflects natural variation in survey completion behavior (such as skipped or unanswered questions). In contrast, the lower level of missingness among suspected bots may indicate automated completion rather than a marker of higher data quality. This difference underscores the importance of evaluating missing data patterns alongside other indicators when assessing data integrity.

Beyond response patterns, the inclusion of bot-generated data introduces a significant risk of bias, particularly in research focusing on health equity. The demographics of suspected bot responses differed notably from those of probable human participants. These patterns may reflect random bot response patterns inflating less common categories; however, this pattern may be partially driven by the ordering of the race or ethnicity response options. In the survey, several minority categories appear in the middle of the list, whereas “White” appears at the bottom. Given the tendency of suspected bots to select middle-of-the-scale responses, the positioning of race or ethnicity response options likely contributed to the higher selection of minority categories among bots. We also acknowledge the possibility that some marginalized respondents were misclassified as suspected bots. This discrepancy poses a major threat to the validity of studies that examine health care use and outcomes among specific demographic subgroups. In equity-focused research, where accurate representation of marginalized populations is crucial, failing to filter out bots could lead to misleading conclusions and inappropriate policy recommendations.

The impact of suspected bot interference is further underscored when examining the relationship between key indicators of depression. Among probable human respondents, the correlation between “having little interest or pleasure in doing things” and “feeling down, depressed, or hopeless” aligned with established patterns in the literature, reflecting a logical association between these 2 related constructs. However, among suspected bot-generated responses, this relationship was unexpectedly reversed, indicating a negative correlation that does not align with theoretical expectations. Notably, a moderate negative correlation suggests a systematic response pattern (such as contradictory response selection) rather than a pattern consistent with genuine symptom co-occurrence. This inconsistency suggests that suspected bot responses do not accurately reflect underlying psychological constructs, reinforcing the need to identify and eliminate suspected bots from survey research. The inconsistency also provides face validity to our bot-detection algorithm.

### Bot Detection Strategies

Several methods to identify suspected bots that were used in this study align with those used in similar research. These include: analyzing open-ended responses for nonsensical, duplicative, or inconsistent data [5,8,10,18]; tracking survey completion times [5,7,8,13,19,20]; flagging surveys that were completed at unusual times of the day [8]; and identifying suspicious patterns related to zip codes and geographic location [20]. We applied all applicable bot detection strategies for which we had data available. There are additional bot detection techniques that were not used in this study but have been used in prior research. These include: checking participants' responses to questions that are asked in 2 ways [8,14,19], (such as “how old are you?” and “what y were you born?”); using IP addresses to check for responses that were completed in the same household or very close to one another in relation to the time the survey was submitted [5,14]; and examining email addresses for suspicious wording or similar content [8,20]. Another strategy involves external validation of participant information, such as verifying names, addresses, phone numbers, and birth dates to ensure responses are unique and legitimate [21]. Investigators can also cross-reference participants’ details using publicly available databases, social media, or professional networking sites to further confirm authenticity, although ethical considerations must be weighed when implementing such approaches [21]. While these strategies may help reduce fraudulent responses, they can also create additional burdens and reduce anonymity for legitimate participants.

Among the bot detection strategies used in this study, some were particularly effective in identifying suspected bot-generated responses. Notably, open-ended responses provided valuable insight, as suspected bot-generated answers often contained repetitive, illogical, or off-topic content across multiple responses. Unusual quantitative responses alone can be difficult to interpret, as they might seem plausible despite being artificially generated. However, open-ended responses can provide valuable context that may help in assessing the authenticity of data. Unlike structured response options, which bots can randomly or systematically select, qualitative responses require coherence and reasoning, making them a powerful tool for distinguishing between genuine participants and automated responses. That said, recent advances in generative artificial intelligence mean that open-text answers are becoming increasingly sophisticated and may no longer be a fully reliable safeguard against fraud [22]. Geographic inconsistencies, such as invalid or duplicate postal codes, or locations outside the intended survey region, were also strong indicators of suspected bot activity, although individual respondents may misreport to preserve anonymity. Additionally, anomalies in time-related metrics, including extremely short survey completion times, completion at unusual hours, or multiple surveys submitted at identical timestamps, further helped differentiate suspected bots from probable human respondents. Based on these findings, we recommend collecting postal or ZIP codes and incorporating multiple timestamps within surveys to enhance suspected bot detection. While no single strategy is universally effective across all surveys, we recommend using a combination of detection methods tailored to the specific survey and target population.

While several strategies are outlined to detect bots, it is also important to prevent bots from entering surveys in the first place. Tools such as Completely Automated Public Turing test to tell Computers and Humans Apart (CAPTCHA) have been developed to offer an additional level of security against bot infiltration [23]. However, research has shown that these tools can be bypassed, as bots are increasingly programmed to recognize and circumvent such challenges [7,20,24]. Similarly, honeypot questions (hidden prompts designed to engage only automated respondents) have also proven ineffective, as more sophisticated bots can now avoid these traps [8,20]. Indeed, researchers who have used this method have reported that bots did not provide responses to these questions [8]. Given the growing complexity of automated survey fraud, it is essential for technology companies to continue developing advanced bot detection solutions. Survey platforms such as REDCap and Qualtrics should integrate these technologies to automate bot prevention, reducing the burden on researchers and ensuring more consistent data quality measures.

### Recommendations

It is recommended that bot detection strategies be implemented at multiple stages in the research process. This includes prior to data collection (eg, using targeted recruitment with specific distribution lists), during data collection (eg, monitoring data daily to identify potential fraud), and following data collection (eg, implementing a systemic check of potential fraud) [18]. Finally, increasing awareness among researchers about the impact of bots in survey research is crucial. We recommend that training in bot prevention and detection be incorporated into research methods and survey design coursework to equip researchers with the necessary skills to safeguard data integrity. There will continue to be a value and need to reach out with surveys to broad groups of public and enabling survey research in this context will continue to be important.

As internet-based research (and survey research in particular) continues to expand, there is a growing opportunity for researchers across disciplines to contribute to the development of comprehensive bot detection methodologies. Moving forward, greater efforts should be directed toward developing universal technologies and initiatives for bot detection. Standardized approaches that can be seamlessly integrated across different research platforms will help safeguard the validity of online data collection and ensure that digital survey research remains a trusted tool for generating meaningful insights.

### Limitations

While this study offers valuable insights, several limitations must be acknowledged. Since bot detection methods were applied to a single case study, their effectiveness has yet to be evaluated across different datasets, which may limit generalizability. Additionally, the data were collected within one region of Ontario, and the characteristics of the probable human responses, and their differences from suspected bot responses, may not be representative of broader populations. As response patterns may vary across settings, researchers working in other regions or with different populations should assess whether similar differences between suspected bot and probable human data are observed. However, the bot identification framework is adaptable, as criteria, such as postal or ZIP code validation, open-text analysis, and completion times can be tailored to reflect local contexts. Differences observed between probable human and suspected bot responses may also partially reflect differences between recruitment channels, such as respondents reached through email lists versus social media postings. Survey respondents recruited via open online platforms may differ systematically in demographics or response behaviors, independent of bot activity. Although some degree of misclassification is therefore possible, our detection framework was deliberately conservative and assumed responses were human unless sufficient evidence across multiple indicators suggested otherwise. Despite using various suspected bot detection strategies, there is also potential for undetected sophisticated bots, which could still impact the dataset’s validity. Furthermore, the study was conducted at a specific point in time, and as automated response technologies evolve, the effectiveness of these detection methods may diminish. The study relied on assumed response patterns to identify suspected bots, but without absolute verification, such as IP tracking or manual validation, there remains some uncertainty in classification. Finally, while we observed that suspected bot responses reversed an expected positive correlation between depression-related items, this pattern was not incorporated into our 3-tier classification framework. Such a pattern would align conceptually with a Tier 3 criterion, as it represents a complex and context-dependent anomaly that would require corroboration from additional indicators to support classification. Future research should examine whether anomalies in response patterns, in addition to structural indicators, can serve as reliable markers of fraudulent responses.

### Conclusions

This study provides criteria for identifying bots in online surveys. In comparing survey results from the suspected bots and probable humans, identified by the developed criteria, we found important differences in response patterns that would jeopardize data validity if bots were not correctly identified. By incorporating robust data validation protocols early in the research process, researchers can minimize bias and prevent the inclusion of inauthentic data, strengthening the reliability of findings. This study should be interpreted as a cautionary case rather than a prescriptive template. Without verified contact lists or other forms of participant validation, open online surveys will remain vulnerable to contamination despite the use of screening methods.

## Supplementary material

10.2196/73622 Multimedia Appendix 1 Ontario Health Team Patient Survey (full survey instrument used in the study).

10.2196/73622 Multimedia Appendix 2 Chi-square test results comparing survey responses between probable humans and suspected bots, with Bonferroni-adjusted *P* values and Cramer V effect sizes.

## Abbreviations

CAPTCHA: Completely Automated Public Turing test to tell Computers and Humans Apart
OHT: Ontario Health Team
PHQ-2: Patient Health Questionnaire-2
PREM: patient-reported experience measure
PROM: patient-reported outcome measure
REDCap: Research Electronic Data Capture
