# Supplementary material for: Identifying and Analyzing Bot-Generated Responses in Online Health Care Surveys: Methodological Study
Source: JMIR Med Inform. 2026 Mar 5;14:e73622. doi: 10.2196/73622 (PMC12978908; doi:10.2196/73622)
Supplement: Multimedia Appendix 1 [file medinform-v14-e73622-s001.docx]

### OHT Patient Survey

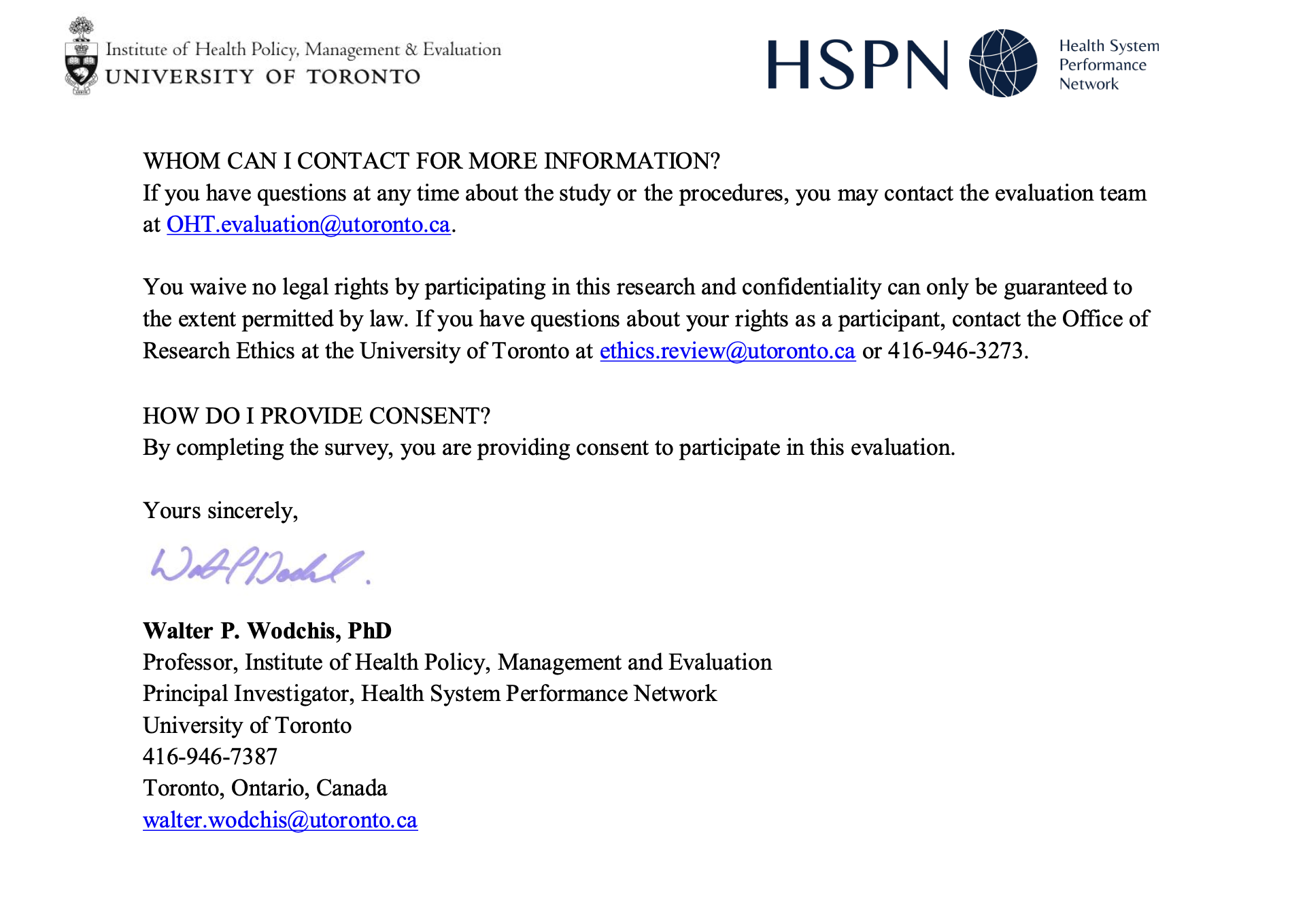


[Timestamp 1]

Have you read and understood the Information Regarding: Ontario Health Team Patient Survey attached to the survey invitation email? *Required

Yes No

[Timestamp 2]

**ONTARIO HEALTH TEAM PATIENT SURVEY**

**Welcome to the Ontario Health Team Patient Survey from the Health System Performance Network. We would love your feedback to help us identify things that can be improved in the delivery of your care and services from your Ontario Health Team (OHT).**

**Your name will not be attached to the responses you give, i.e., you will remain anonymous. Your results will be combined with those of other respondents to provide an overall assessment of the experience of patients cared for within OHTs and to identify areas where patient experience can be improved. The results will only be shared with the OHT when there are at least 10 respondents.**

**The survey will take approximately 15 minutes to complete. Please select the answer that best describes you and your experiences.**

**If you need any help, please contact the person who sent you this survey. You can get a friend or caregiver to help you complete this survey as well.**

**Thank you for taking the time to complete this survey.**

In the last 12 months, have you received care or community services in any of the following areas: [name of areas]*

Yes

No

What are the FIRST THREE digits of your postal code?

* __________________________________

(i.e., A1A)

**SECTION A: HEALTHCARE USE**

In the last 12 months, who did you receive care from? *

)

Select all that apply.

(

Primary care physician

Nurse practitioner

Specialist physician or Hospital outpatient clinic

Mental health services

Home care nursing or Rehabilitation

Home care personal support / Homemaking

EMS / Ambulance services

Emergency Department

Hospital with overnight stay

Allied health professionals (e.g., physio / occupational / respiratory therapist, social worker, etc.)

Pharmacy

Social or community programs and services (e.g., community health worker, supports for daily living, adult

day program, etc.)

Caregiver respite services

Housing support (including supportive housing)

Voluntary services (e.g., Meals on Wheels)

Other (Please specify.) ______

I don't know. / I don't remember.

How often do you interact with the health system? *

(This could include home care, community supports, primary care, etc.)

Multiple times a week

Weekly

Monthly

A few times a year

Annually

Other (Please specify.) _____

**SECTION B: YOUR HEALTH We would like to know what you think about your health.**

In general, how would you describe your own health? *

Excellent

Very good

Good

Fair

Poor

I don't know.

Please choose an answer that best describes your mobility today. *

I have no problems walking about.

I have slight problems walking about.

I have moderate problems walking about.

I have severe problems walking about.

I am unable to walk about.

Please choose an answer that best describes your self-care today. *

I have no problems washing or dressing myself.

I have slight problems washing or dressing myself.

I have moderate problems washing or dressing myself.

I have severe problems washing or dressing myself.

I am unable to wash or dress myself.

Please choose an answer that best describes your usual activities today. * These could be work, studying, housework, family, or leisure activities.

I have no problems doing my usual activities.

I have slight problems doing my usual activities.

I have moderate problems doing my usual activities.

I have severe problems doing my usual activities.

I am unable to do my usual activities.

Please choose an answer that best describes your level of pain or discomfort today. *

I have no pain or discomfort.

I have slight pain or discomfort.

I have moderate pain or discomfort.

I have severe pain or discomfort.

I have extreme pain or discomfort.

Please choose an answer that best describes your level of anxiety or depression today. *

I am not anxious or depressed.

I am slightly anxious or depressed.

I am moderately anxious or depressed.

I am severely anxious or depressed.

I am extremely anxious or depressed.

Over the past two weeks, how often have you been bothered by any of the following

**problems?**

Not at all

Several days (less

than half the days)

More than one-half

the days

Nearly every day

Little interest or pleasure in

doing things *

Feeling down, depressed, or

hopeless *

Substance use is different for everyone and can be viewed on a spectrum with varying stages of benefits and harms.

Which stage is most accurate in your situation? *

Non-use (Abstinence, i.e., not using drugs, tobacco or alcohol.)

Beneficial use (Beneficial use is use that can have positive health, social, or spiritual effects, e.g., taking

medication as prescribed, ceremonial/religious use of tobacco, such as smudging, etc.)

Lower risk use (Lower-risk use has minimal impact on a person, their family, friends and others, e.g., drinking following the low-risk alcohol drinking guidelines, using cannabis according to the lower-risk cannabis use guidelines, etc.)

Higher-risk use (Higher-risk use is use that has a harmful and negative impact to a person, their family, friends and others, e.g., binge drinking, impaired driving, use of illegal drugs, etc.)

Addiction (Substance use disorder, i.e., when someone cannot stop using drugs, tobacco or alcohol even if they want to.)

**SECTION C: EASILY ACCESSING CARE**

Do you have a health professional that you see for regular check-ups when you are sick, and so on? * This could be a family doctor, a general practitioner or GP, or a nurse practitioner.

Note: In this survey, we refer to this individual as your "regular healthcare provider".

Yes

No

I don't know.

In the last 12 months, how would you describe the length of time it took to access your regular healthcare provider?

*

About right

Somewhat too long

Much too long

I did not see my regular healthcare provider in last 12 months.

I don't know. / I don't remember.

Sometimes, in order to maintain their health, people need to have help with meal preparation, transportation, housework, laundry, and so on (we call this "community supports").

Do you ever need this type of help? *

Yes

No

I don't know.

In general, how easy is it for you to get community supports? *

Very easy

Somewhat easy

Somewhat difficult

Very difficult

I don't know. / I don't remember.

In the last 12 months, how would you describe the length of time it took to get community supports? *

About right

Somewhat too long

Much too long

I did not need community supports in last 12 months.

I don't know. / I don't remember.

When you consider how you and all your healthcare providers help you take care of your health, how coordinated would you say your overall healthcare is? *

[Timestamp 3]

Very coordinated

Somewhat coordinated

Not coordinated

I don't know.

__________________________________

SECTION D: HAVING SOMEONE TO COUNT ON

In general, how confident are you that your regular healthcare provider or other healthcare professional checks to make sure you receive the healthcare you need? *

Very confident

Somewhat confident

Not very confident

Not at all confident

I don't know.

Is there at least one person, other than a healthcare professional, who helps make sure you receive the healthcare you need? *

This could be a family member, friend, or someone else.

Yes

No

I am able to take care of myself.

I don't know.

How confident are you that this person will look after you as you get older or as your health changes? *

Very confident

Somewhat confident

Not very confident

Not at all confident

I don't know.

Do you identify as a caregiver for someone else? *

A caregiver is a family member, partner or friend who, regularly and without pay, provides support or care to a person who has health needs.

Yes No

I don't know.

SECTION E: BEING HEARD

When you see your regular healthcare provider or someone else in their office, how often do they involve you as much as you want to be in decisions about your care and treatment? *

Always

Often

Sometimes

Rarely

Never

I don't know.

In general, how well do you feel your healthcare providers understand your healthcare needs? *

Very well

Somewhat well

Not very well

Not at all well

I don't know.

In general, would you say your healthcare providers listen carefully to you? *

Always

Often

Sometimes

Rarely

Never

I don't know.

In general, do your healthcare providers encourage you to bring someone with you to your appointments? *

Always

Often

Sometimes

Rarely

Never

Not Applicable / I don't know.

SECTION F: KNOWING HOW TO MANAGE HEALTH

In general, how confident are you that you know the things that you need to do to take care of and manage your health? *

Very confident

Somewhat confident

Not very confident

Not at all confident

I don't know.

In the last 12 months, was there ever a time when you received conflicting information about your healthcare from different healthcare providers such as your family doctor, specialists, or other healthcare providers including nurses, dieticians, staff at clinics, and so on? *

Yes No

I don't know. / I don't remember.

SECTION G: SAFETY

Are you able to move around your home and neighbourhood without fear of falling or getting disoriented? *

Yes

No

I don't know.

[Timestamp 4]

SECTION H: TRANSITIONS

EMERGENCY

In the last 12 months, have you been to an emergency department (ED) because you were sick or for a health-related problem? *

Yes

No

I don't know. / I don't remember.

Did you have to return to the ED for the SAME REASON that you either visited an ED or were hospitalized previously for? *

Yes

No

I don't know. / I don't remember.

The last time you went to the ED, was it for a condition that you think could have been treated by your regular healthcare provider or other healthcare professional if he/she had been available? *

Very confident

Somewhat confident

Not very confident

Not at all confident

I don't know. / I don't remember.

HOSPITAL

In the last 12 months, have you been hospitalized overnight? *

Yes

No

I don't know. / I don't remember.

Yes

No

I don't know. / I don't remember.

The last time you went to the ED, which of the following was the MAIN reason you went to the ED rather than to your regular healthcare provider or another healthcare professional? *

It was an emergency

My provider was not available.

I could not get an appointment with my provider.

It was faster to go to the emergency.

The ED was closer.

My provider advised me to go to the ED.

My regular healthcare provider works out of ED.

Other (Please specify.) ______

I don't know. / I don't remember.

The last time you went to the ED, when you left, how confident were you that you had the information you needed to care for and manage the health problem for which you went to the ED?

When you left the hospital, were you provided with easy-to-follow instructions on whom to contact if you had a question about your treatment or if your condition became worse? *

Yes

No

I don't know. / I don't remember.

After you were discharged from hospital, did your regular healthcare provider or other healthcare professional seem informed and up-to-date about the care you received in the hospital? *

Yes

No

I have not seen my regular provider or other healthcare professionals since being discharged from hospital.

I don't know. / I don't remember.

SPECIALISTS

In the last 12 months, have you seen a medical specialist? *

This includes an appointment in person, by phone, video, email, or secure message.

Yes

No

I don't know. / I don't remember.

How would you rate the length of time it took between making the appointment and the actual visit? *

About right

Somewhat too long

Much too long

I don't know. / I don't remember.

When you last saw the specialist, did he/she have basic medical information from your regular healthcare provider about the reason for your visit? *

Yes

No

I don't know. / I don't remember.

After you saw the specialist, did your regular healthcare provider seem informed and up-to-date about the care you got from the specialist? *

Yes

No

I don't know. / I don't remember.

Has your health gotten worse because you were not able to access a specialist? *

Yes

No

Not Applicable / I have not had the need to see a specialist.

TESTS

In the last 12 months, when receiving care for a medical problem, was there ever a time when test results were not available at the time of a scheduled appointment with your provider? *

Yes No

Not Applicable / I did not have any tests in the last 12 months. I don't know. / I don't remember.

SECTION I: DIGITAL HEALTHCARE

Doctors keep medical records of their patients which includes information such as age, weight, clinical notes from appointments, existing medical conditions, etc. Some physicians allow their patients to have online access so that patients can see their medical records outside of the doctor's office. Some examples of these systems are MyChart, MyUHN, etc.

In the last 12 months, have you looked at your medical records using an online portal or digital tool? *

This does not include being able to access results of lab tests completed at labs such as Lifelabs or Dynacare and provided by the lab.

Yes

No

I don't know. / I don't remember.

Which of the following is the MAIN reason you have not looked at your medical records online?

I did not want to check my medical records this way.

My provider does not make medical records available this way.

I do not know how to.

I do not have reliable/any access to the internet.

I do not have reliable/any access to tools needed (including computer, laptop, tablet, etc.) I had no need to look at my medical records.

I never knew you could do this. I don't know.

In the last 12 months, have you looked at your medical or health records using online portals or digital tools that are designed for people with specific health conditions? * An example of this type of tool is NED or Medly.

Yes

No

I don't know. / I don't remember.

Other than for booking an appointment, in the last 12 months, have you used any of the following types of thods to communicate with your regular healthcare provider or other healthcare professional about your medical care? *

(

Select all that apply.

)

Telephone

Email

Website or portal

Video call

Text messaging / Electronic messaging

Other method(s) (Please specify.) ______

Not Applicable / I did not communicate with any healthcare provider virtually.

I don't know. / I don't remember.

__________________________________

[Timestamp 5]

**ADDITIONAL FEEDBACK**

Do you have any suggestions for how your healthcare experience can be improved?

SECTION J: ABOUT YOU

How old are you?

Under 18 years old

18-24

years old

25-44

years old

45-64

years old

65-74

years old

75-84

years old

85

years old or older

Prefer not to answer

Select the gender category you identify with.

Woman

Man

Trans woman

Trans man

Two-Spirit

Another gender identity (Please specify.) ______

Prefer not to answer

Select the sexual orientation you identify with.

Bisexual

Heterosexual (Straight)

Homosexual (Gay/Lesbian)

Queer

Two-Spirit

Another sexual orientation (Please specify.) ______

Prefer not to answer

Select the race(s)/ethnicity(ies) you identify with.

(Select all that apply.)

Asian - East (e.g., Chinese, Japanese, Korean)

Asian - South East (e.g., Malaysian, Filipino, Vietnamese)

Asian - South (e.g., Indian, Pakistani, Sri Lankan)

Indo-Caribbean (e.g., Guyanese with origins in India)

Middle Eastern / North African (e.g., Algerian, Iranian, Lebanese)

Black - Sub-Saharan African (e.g., Ghanaian, Kenyan, Somali)

Black - North American (e.g., Canadian, American)

Black - Caribbean (e.g., Barbadian, Jamaican)

Latin American / Hispanic (e.g., Argentinean, Chilean, Salvadoran)

First Nations (Status/Non-Status Indian)

Inuk/Inuit

Métis

White - European (e.g., English, Italian, Russian)

White - North American (e.g., Canadian, American)

You do not have an option that applies to me. (Please specify.) ______ Prefer not to answer.

When you see or speak with nurses, doctors, physicians, specialists, and others in the healthcare system, in what official language are you most comfortable?

English

French

I am most comfortable in another language. (Please specify.) ______

The next questions ask about how you feel in your current living situation.

Do you have difficulty paying all your bills at the end of the month?

Always

Sometimes

Rarely

Never

Prefer not to answer

After paying your monthly bills, do you typically have enough money left for food?

Always

Sometimes

Rarely

Never

Prefer not to answer

Do you ever worry about losing your place to live?

Always

Sometimes

Rarely

Never

Prefer not to answer

How often do you feel isolated from others?

Always

Sometimes

Rarely

Never

Prefer not to answer

How often do you feel left out?

Always

Sometimes

Rarely

Never

Prefer not to answer

How often do you feel that you lack companionship?

Always

Sometimes

Rarely

Never

Prefer not to answer

[Timestamp 6]

What type of housing are you currently living in?

Renting

Homeowner

Long-term care

Retirement home / Residence

Affordable Housing Programs (Subsidized housing, Rent-Geared-to-Income (RGI) housing, Rent supplement

programs)

Domiciliary hospital

No current living arrangements (Person who is homeless, couch surfing, etc.)

Other (Please specify.) ______

Who was/were the main person or people that filled in this questionnaire?

Me, the patient/client

A friend or relative of the patient/client

Both me, the patient/client, and a friend or relative, together

Me, the patient/client, with the help of a health professional

__________________________________

Thank you for participating in the survey conducted by the [OHT NAME]. We appreciate your time and valuable input. Enter for a chance to win a gift card! 1 in 40 participants will be selected to win. Participation in the draw is optional.

Enter here: <https://forms.office.com/r/5FaLArHDdP>
