# Supplementary material for: Identifying and Analyzing Bot-Generated Responses in Online Health Care Surveys: Methodological Study
Source: JMIR Med Inform. 2026 Mar 5;14:e73622. doi: 10.2196/73622 (PMC12978908; doi:10.2196/73622)
Supplement: Multimedia Appendix 2 [file medinform-v14-e73622-s002.docx]

**Supplementary Table**: **Chi-square test results comparing survey responses between probable humans and suspected bots, with Bonferroni-adjusted P-values and Cramer’s V effect sizes**

| **Question** | **Level** | **Overall N (%)** | **Probable Human**  **N (%)** | **Suspected Bot**  **N (%)** | **Chi square** | **df** | **Uncorrected p-value** | **Bonferroni-adjusted p-value** | **Cramers V (95% CI)** |
| --- | --- | --- | --- | --- | --- | --- | --- | --- | --- |
| In the last 12 months, who did you receive care from?* | Allied health | 14 (1.3%) | 5 (0.8%) | 9 (1.42%) | 72.43 | 16 | 1.00×10⁻⁵ | 5.90×10⁻⁴ | 0.26 (0.2, 0.32) |
|  | Community | 18 (1.7%) | 2 (0.5%) | 16 (2.53%) |  |  |  |  |  |
|  | ED | 27 (2.5%) | 18 (4.1%) | 9 (1.42%) |  |  |  |  |  |
|  | EMS | 8 (0.7%) | 1 (0.2%) | 7 (1.11%) |  |  |  |  |  |
|  | Home care | 13 (1.2%) | 0 (0.0%) | 13 (2.05%) |  |  |  |  |  |
|  | Hospitalization | 13 (1.2%) | 4 (0.9%) | 9 (1.42%) |  |  |  |  |  |
|  | Housing | 3 (0.3%) | 0 (0.0%) | 3 (0.47%) |  |  |  |  |  |
|  | Mental Health Services | 30 (2.8%) | 6 (1.4%) | 24 (3.79%) |  |  |  |  |  |
|  | Nurse Practitioner | 55 (5.1%) | 26 (5.9%) | 29 (4.58%) |  |  |  |  |  |
|  | Nursing or Rehab | 31 (2.9%) | 1 (0.2%) | 30 (4.74%) |  |  |  |  |  |
|  | Pharmacy | 93 (8.7%) | 35 (8.0%) | 58 (9.16%) |  |  |  |  |  |
|  | Respite | 2 (0.2%) | 0 (0.0%) | 2 (0.32%) |  |  |  |  |  |
|  | Specialist | 54 (5.0%) | 25 (5.7%) | 29 (4.58%) |  |  |  |  |  |
|  | Voluntary | 4 (0.4%) | 0 (0.0%) | 4 (0.63%) |  |  |  |  |  |
|  | Multiple Physicians | 697 (65.1%) | 306 (70.0%) | 391 (61.77%) |  |  |  |  |  |
|  | I don't know | 3 (0.3%) | 3 (0.7%) | 0 (0.00%) |  |  |  |  |  |
|  | Other | 5 (0.5%) | 5 (1.1%) | 0 (0.00%) |  |  |  |  |  |
|  | **Total** | **1070 (100%)** | **437 (100%)** | **633 (100%)** |  |  |  |  |  |
| How often do you interact with the health system? (This could include home care community supports primary care etc)* | A few times a year | 482 (42.1%) | 257 (53.3%) | 225 (33.99%) | 148.80 | 5 | 2.41×10⁻³⁰ | 1.42×10⁻²⁸ | 0.36 (0.31, 0.41) |
|  | Multiple times a week | 104 (9.1%) | 25 (5.2%) | 79 (11.93%) |  |  |  |  |  |
|  | Weekly | 213 (18.6%) | 38 (7.9%) | 175 (26.44%) |  |  |  |  |  |
|  | Monthly | 224 (19.6%) | 74 (15.4%) | 150 (22.66%) |  |  |  |  |  |
|  | Annually | 102 (8.9%) | 70 (14.5%) | 32 (4.83%) |  |  |  |  |  |
|  | Other (Please specify.) | 19 (1.7%) | 18 (3.7%) | 1 (0.15%) |  |  |  |  |  |
|  | **Total** | **1144 (100%)** | **482 (100%)** | **662 (100%)** |  |  |  |  |  |
| In general how would you describe your own health? | Excellent | 111 (9.9%) | 46 (9.9%) | 65 (9.92%) | 19.94 | 5 | 9.50×10⁻⁴ | 5.60×10⁻² | 0.13 (0.08, 0.19) |
|  | Very good | 312 (27.9%) | 144 (31.0%) | 168 (25.65%) |  |  |  |  |  |
|  | Good | 467 (41.7%) | 164 (35.3%) | 303 (46.26%) |  |  |  |  |  |
|  | Fair | 169 (15.1%) | 74 (15.9%) | 95 (14.50%) |  |  |  |  |  |
|  | Poor | 50 (4.5%) | 31 (6.7%) | 19 (2.90%) |  |  |  |  |  |
|  | I don't know. | 10 (0.9%) | 5 (1.1%) | 5 (0.76%) |  |  |  |  |  |
|  | **Total** | **1119 (100%)** | **464 (100%)** | **655 (100%)** |  |  |  |  |  |
| Please choose an answer that best describes your mobility today.* | I have no problems walking about. | 554 (49.5%) | 309 (66.6%) | 245 (37.40%) | 106.03 | 4 | 5.11×10⁻²² | 3.02×10⁻²⁰ | 0.31 (0.25, 0.36) |
|  | I have slight problems walking about. | 313 (28.0%) | 67 (14.4%) | 246 (37.56%) |  |  |  |  |  |
|  | I have moderate problems walking about. | 178 (15.9%) | 57 (12.3%) | 121 (18.47%) |  |  |  |  |  |
|  | I have severe problems walking about. | 52 (4.6%) | 20 (4.3%) | 32 (4.89%) |  |  |  |  |  |
|  | I am unable to walk about. | 22 (2.0%) | 11 (2.4%) | 11 (1.68%) |  |  |  |  |  |
|  | **Total** | **1119 (100%)** | **464 (100%)** | **655 (100%)** |  |  |  |  |  |
| Please choose an answer that best describes your self care today.* | I have no problems washing or dressing myself. | 681 (60.9%) | 396 (85.3%) | 285 (43.51%) | 208.47 | 4 | 5.68×10⁻⁴⁴ | 3.35×10⁻⁴² | 0.43 (0.38, 0.48) |
|  | I have slight problems washing or dressing myself. | 237 (21.2%) | 30 (6.5%) | 207 (31.60%) |  |  |  |  |  |
|  | I have moderate problems washing or dressing myself. | 136 (12.2%) | 21 (4.5%) | 115 (17.56%) |  |  |  |  |  |
|  | I have severe problems washing or dressing myself. | 43 (3.8%) | 7 (1.5%) | 36 (5.50%) |  |  |  |  |  |
|  | I am unable to wash or dress myself. | 22 (2.0%) | 10 (2.2%) | 12 (1.83%) |  |  |  |  |  |
|  | **Total** | **1119 (100%)** | **464 (100%)** | **655 (100%)** |  |  |  |  |  |
| Please choose an answer that best describes your usual activities today. (These could be work, studying, housework, family or leisure activities).* | I have no problems doing my usual activities. | 590 (52.7%) | 293 (63.1%) | 297 (45.34%) | 40.20 | 4 | 3.93×10⁻⁸ | 2.32×10⁻⁶ | 0.19 (0.13, 0.25) |
|  | I have slight problems doing my usual activities. | 329 (29.4%) | 94 (20.3%) | 235 (35.88%) |  |  |  |  |  |
|  | I have moderate problems doing my usual activities. | 144 (12.9%) | 55 (11.9%) | 89 (13.59%) |  |  |  |  |  |
|  | I have severe problems doing my usual activities. | 40 (3.6%) | 17 (3.7%) | 23 (3.51%) |  |  |  |  |  |
|  | I am unable to do my usual activities. | 16 (1.4%) | 5 (1.1%) | 11 (1.68%) |  |  |  |  |  |
|  | **Total** | **1119 (100%)** | **464 (100%)** | **655 (100%)** |  |  |  |  |  |
| Please choose an answer that best describes your level of pain or discomfort today. | I have no pain or discomfort. | 391 (34.9%) | 157 (33.8%) | 234 (35.73%) | 2.40 | 4 | .662 | 1.00 | 0.05 (0.0, 0.1) |
|  | I have slight pain or discomfort. | 431 (38.5%) | 185 (39.9%) | 246 (37.56%) |  |  |  |  |  |
|  | I have moderate pain or discomfort. | 216 (19.3%) | 87 (18.8%) | 129 (19.69%) |  |  |  |  |  |
|  | I have severe pain or discomfort. | 61 (5.5%) | 24 (5.2%) | 37 (5.65%) |  |  |  |  |  |
|  | I have extreme pain or discomfort. | 20 (1.8%) | 11 (2.4%) | 9 (1.37%) |  |  |  |  |  |
|  | **Total** | **1119 (100%)** | **464 (100%)** | **655 (100%)** |  |  |  |  |  |
| Please choose an answer that best describes your level of anxiety or depression today. | I am not anxious or depressed. | 431 (38.5%) | 178 (38.4%) | 253 (38.63%) | 16.45 | 4 | 2.48×10⁻³ | 0.146 | 0.12 (0.06, 0.18) |
|  | I am slightly anxious or depressed. | 453 (40.5%) | 171 (36.9%) | 282 (43.05%) |  |  |  |  |  |
|  | I am moderately anxious or depressed. | 163 (14.6%) | 73 (15.7%) | 90 (13.74%) |  |  |  |  |  |
|  | I am severely anxious or depressed. | 50 (4.5%) | 25 (5.4%) | 25 (3.82%) |  |  |  |  |  |
|  | I am extremely anxious or depressed. | 22 (2.0%) | 17 (3.7%) | 5 (0.76%) |  |  |  |  |  |
|  | **Total** | **1119 (100%)** | **464 (100%)** | **655 (100%)** |  |  |  |  |  |
| Over the past two weeks, how often have you been bothered by any of the following problems?: Little interest or pleasure in doing things* | Not at all | 364 (32.9%) | 233 (51.7%) | 131 (20.00%) | 157.20 | 3 | 7.36×10⁻³⁴ | 4.34×10⁻³² | 0.38 (0.32, 0.43) |
|  | Several days (less than half the days) | 496 (44.8%) | 146 (32.4%) | 350 (53.44%) |  |  |  |  |  |
|  | More than one-half the days | 182 (16.5%) | 33 (7.3%) | 149 (22.75%) |  |  |  |  |  |
|  | Nearly every day | 64 (5.8%) | 39 (8.6%) | 25 (3.82%) |  |  |  |  |  |
|  | **Total** | **1106 (100%)** | **451 (100%)** | **655 (100%)** |  |  |  |  |  |
| Over the past two weeks, how often have you been bothered by any of the following problems?: Feeling down depressed or hopeless* | Not at all | 444 (40.1%) | 235 (52.1%) | 209 (31.91%) | 87.05 | 3 | 9.40×10⁻¹⁹ | 5.54×10⁻¹⁷ | 0.28 (0.22, 0.34) |
|  | Several days (less than half the days) | 395 (35.7%) | 145 (32.2%) | 250 (38.17%) |  |  |  |  |  |
|  | More than one-half the days | 209 (18.9%) | 36 (8.0%) | 173 (26.41%) |  |  |  |  |  |
|  | Nearly every day | 58 (5.2%) | 35 (7.8%) | 23 (3.51%) |  |  |  |  |  |
|  | **Total** | **1106 (100%)** | **451 (100%)** | **655 (100%)** |  |  |  |  |  |
| Substance use is different for everyone and can be viewed on a spectrum with varying stages of benefits and harms. Which stage is most accurate in your situation?* | Non-use (Abstinence, i.e., not using drugs, tobacco or alcohol.) | 299 (27.0%) | 176 (39.0%) | 123 (18.78%) | 98.07 | 4 | 2.53×10⁻²⁰ | 1.49×10⁻¹⁸ | 0.30 (0.24, 0.35) |
|  | Beneficial use (Beneficial use is use that can have positive health, social, or spiritual effects, e.g., taking medication as prescribed, ceremonial/religious use of tobacco, such as smudging, etc.) | 419 (37.9%) | 117 (25.9%) | 302 (46.11%) |  |  |  |  |  |
|  | Lower risk use (Lower-risk use has minimal impact on a person, their family, friends and others, e.g., drinking following the low-risk alcohol drinking guidelines, using cannabis according to the lower-risk cannabis use guidelines, etc.) | 299 (27.0%) | 139 (30.8%) | 160 (24.43%) |  |  |  |  |  |
|  | Higher-risk use (Higher-risk use is use that has a harmful and negative impact to a person, their family, friends and others, e.g., binge drinking, impaired driving, use of illegal drugs, etc.) | 68 (6.1%) | 8 (1.8%) | 60 (9.16%) |  |  |  |  |  |
|  | Addiction (Substance use disorder, i.e., when someone cannot stop using drugs, tobacco or alcohol even if they want to.) | 21 (1.9%) | 11 (2.4%) | 10 (1.53%) |  |  |  |  |  |
|  | **Total** | **1106 (100%)** | **451 (100%)** | **655 (100%)** |  |  |  |  |  |
| Do you have a health professional that you see for regular check-ups when you are sick, and so on? This could be a family doctor, a general practitioner or GP, or a nurse practitioner.* | Yes | 810 (74.0%) | 379 (85.4%) | 431 (66.21%) | 60.69 | 2 | 6.62×10⁻¹⁴ | 3.90×10⁻¹² | 0.24 (0.18, 0.29) |
|  | No | 230 (21.0%) | 63 (14.2%) | 167 (25.65%) |  |  |  |  |  |
|  | I don't know. | 55 (5.0%) | 2 (0.5%) | 53 (8.14%) |  |  |  |  |  |
|  | **Total** | **1095 (100%)** | **444 (100%)** | **651 (100%)** |  |  |  |  |  |
| In the last 12 months how would you describe the length of time it took to access your regular healthcare provider?* | About right | 304 (37.5%) | 197 (52.0%) | 107 (24.83%) | 84.51 | 4 | 1.92×10⁻¹⁷ | 1.13×10⁻¹⁵ | 0.32 (0.26, 0.39) |
|  | Somewhat too long | 330 (40.7%) | 97 (25.6%) | 233 (54.06%) |  |  |  |  |  |
|  | Much too long | 122 (15.1%) | 60 (15.8%) | 62 (14.39%) |  |  |  |  |  |
|  | I did not see my regular healthcare provider in last 12 months. | 36 (4.4%) | 13 (3.4%) | 23 (5.34%) |  |  |  |  |  |
|  | I don't know. / I don't remember. | 18 (2.2%) | 12 (3.2%) | 6 (1.39%) |  |  |  |  |  |
|  | **Total** | **810 (100%)** | **379 (100%)** | **431 (100%)** |  |  |  |  |  |
| Sometimes, in order to maintain their health, people need to have help with meal preparation, transportation, housework, laundry, and so on (we call this "community supports").Do you ever need this type of help?* | Yes | 419 (38.3%) | 72 (16.2%) | 347 (53.30%) | 197.09 | 2 | 1.60×10⁻⁴³ | 9.42×10⁻⁴² | 0.42 (0.37, 0.48) |
|  | No | 619 (56.5%) | 364 (82.0%) | 255 (39.17%) |  |  |  |  |  |
|  | I don't know. | 57 (5.2%) | 8 (1.8%) | 49 (7.53%) |  |  |  |  |  |
|  | **Total** | **1095 (100%)** | **444 (100%)** | **651 (100%)** |  |  |  |  |  |
| In general how easy is it for you to get community supports? * | Very easy | 46 (11.0%) | 15 (20.8%) | 31 (8.93%) | 61.30 | 4 | 1.00×10⁻⁵ | 5.90×10⁻⁴ | 0.38 (0.29, 0.47) |
|  | Somewhat easy | 160 (38.2%) | 14 (19.4%) | 146 (42.07%) |  |  |  |  |  |
|  | Somewhat difficult | 174 (41.5%) | 21 (29.2%) | 153 (44.09%) |  |  |  |  |  |
|  | Very difficult | 36 (8.6%) | 20 (27.8%) | 16 (4.61%) |  |  |  |  |  |
|  | I don't know. / I don't remember. | 3 (0.7%) | 2 (2.8%) | 1 (0.29%) |  |  |  |  |  |
|  | **Total** | **419 (100%)** | **72 (100%)** | **347 (100%)** |  |  |  |  |  |
| In the last 12 months, how would you describe the length of time it took to get community supports? * | About right | 146 (34.8%) | 27 (37.5%) | 119 (34.29%) | 50.50 | 4 | 1.00×10⁻⁵ | 5.90×10⁻⁴ | 0.35 (0.26, 0.44) |
|  | Somewhat too long | 177 (42.2%) | 13 (18.1%) | 164 (47.26%) |  |  |  |  |  |
|  | Much too long | 72 (17.2%) | 20 (27.8%) | 52 (14.99%) |  |  |  |  |  |
|  | I did not need community supports in last 12 months. | 11 (2.6%) | 2 (2.8%) | 9 (2.59%) |  |  |  |  |  |
|  | I don't know. / I don't remember. | 13 (3.1%) | 10 (13.9%) | 3 (0.86%) |  |  |  |  |  |
|  | **Total** | **419 (100%)** | **72 (100%)** | **347 (100%)** |  |  |  |  |  |
| When you consider how you and all your healthcare providers help you take care of your health, how coordinated would you say your overall healthcare is? * | Very coordinated | 291 (26.6%) | 125 (28.2%) | 166 (25.50%) | 61.33 | 3 | 3.06×10⁻¹³ | 1.81×10⁻¹¹ | 0.24 (0.18, 0.29) |
|  | Somewhat coordinated | 604 (55.2%) | 194 (43.7%) | 410 (62.98%) |  |  |  |  |  |
|  | Not coordinated | 164 (15.0%) | 107 (24.1%) | 57 (8.76%) |  |  |  |  |  |
|  | I don't know. | 36 (3.3%) | 18 (4.1%) | 18 (2.76%) |  |  |  |  |  |
|  | **Total** | **1095 (100%)** | **444 (100%)** | **651 (100%)** |  |  |  |  |  |
| In general, how confident are you that your regular healthcare provider or other healthcare professional checks to make sure you receive the healthcare you need?* | Very confident | 238 (22.0%) | 118 (26.9%) | 120 (18.58%) | 29.43 | 4 | 6.39×10⁻⁶ | 3.77×10⁻⁴ | 0.16 (0.11, 0.22) |
|  | Somewhat confident | 525 (48.4%) | 172 (39.3%) | 353 (54.64%) |  |  |  |  |  |
|  | Not very confident | 198 (18.3%) | 83 (18.9%) | 115 (17.80%) |  |  |  |  |  |
|  | Not at all confident | 104 (9.6%) | 56 (12.8%) | 48 (7.43%) |  |  |  |  |  |
|  | I don't know. | 19 (1.8%) | 9 (2.1%) | 10 (1.55%) |  |  |  |  |  |
|  | **Total** | **1084 (100%)** | **438 (100%)** | **646 (100%)** |  |  |  |  |  |
| Is there at least one person other than a healthcare professional who helps make sure you receive the healthcare you need? This could be a family member, friend or someone else.* | Yes | 524 (48.3%) | 178 (40.6%) | 346 (53.56%) | 101.52 | 3 | 7.34×10⁻²² | 4.33×10⁻²⁰ | 0.31 (0.25, 0.36) |
|  | No | 281 (25.9%) | 88 (20.1%) | 193 (29.88%) |  |  |  |  |  |
|  | I am able to take care of myself. | 243 (22.4%) | 165 (37.7%) | 78 (12.07%) |  |  |  |  |  |
|  | I don't know. | 36 (3.3%) | 7 (1.6%) | 29 (4.49%) |  |  |  |  |  |
|  | **Total** | **1084 (100%)** | **438 (100%)** | **646 (100%)** |  |  |  |  |  |
| How confident are you that this person will look after you as you get older or as your health changes? * | Very confident | 225 (43.0%) | 98 (55.4%) | 127 (36.71%) | 20.92 | 4 | 2.10×10⁻⁴ | 0.012 | 0.20 (0.12, 0.28) |
|  | Somewhat confident | 240 (45.9%) | 61 (34.5%) | 179 (51.73%) |  |  |  |  |  |
|  | Not very confident | 47 (9.0%) | 12 (6.8%) | 35 (10.12%) |  |  |  |  |  |
|  | Not at all confident | 8 (1.5%) | 4 (2.3%) | 4 (1.16%) |  |  |  |  |  |
|  | I don't know. | 3 (0.6%) | 2 (1.1%) | 1 (0.29%) |  |  |  |  |  |
|  | **Total** | **523 (100%)** | **177 (100%)** | **346 (100%)** |  |  |  |  |  |
| Do you identify as a caregiver for someone else? * | Yes | 603 (55.7%) | 173 (39.6%) | 430 (66.56%) | 113.06 | 2 | 2.81×10⁻²⁵ | 1.66×10⁻²³ | 0.32 (0.27, 0.38) |
|  | No | 432 (39.9%) | 257 (58.8%) | 175 (27.09%) |  |  |  |  |  |
|  | I don't know. | 48 (4.4%) | 7 (1.6%) | 41 (6.35%) |  |  |  |  |  |
|  | **Total** | **1083 (100%)** | **437 (100%)** | **646 (100%)** |  |  |  |  |  |
| When you see your regular healthcare provider or someone else in their office, how often do they involve you as much as you want to be in decisions about your care and treatment? * | Always | 280 (26.1%) | 192 (44.9%) | 88 (13.62%) | 162.34 | 5 | 3.14×10⁻³³ | 1.85×10⁻³¹ | 0.39 (0.33, 0.44) |
|  | Often | 281 (26.2%) | 107 (25.0%) | 174 (26.93%) |  |  |  |  |  |
|  | Rarely | 99 (9.2%) | 33 (7.7%) | 66 (10.22%) |  |  |  |  |  |
|  | Sometimes | 344 (32.0%) | 65 (15.2%) | 279 (43.19%) |  |  |  |  |  |
|  | Never | 36 (3.4%) | 17 (4.0%) | 19 (2.94%) |  |  |  |  |  |
|  | I don't know. | 34 (3.2%) | 14 (3.3%) | 20 (3.10%) |  |  |  |  |  |
|  | **Total** | **1074 (100%)** | **428 (100%)** | **646 (100%)** |  |  |  |  |  |
| In general, how well do you feel your healthcare providers understand your healthcare needs? * | Very well | 249 (23.2%) | 161 (37.6%) | 88 (13.62%) | 84.71 | 4 | 1.75×10⁻¹⁷ | 1.03×10⁻¹⁵ | 0.28 (0.22, 0.34) |
|  | Somewhat well | 498 (46.4%) | 167 (39.0%) | 331 (51.24%) |  |  |  |  |  |
|  | Not very well | 214 (19.9%) | 64 (15.0%) | 150 (23.22%) |  |  |  |  |  |
|  | Not at all well | 82 (7.6%) | 28 (6.5%) | 54 (8.36%) |  |  |  |  |  |
|  | I don't know. | 31 (1.9%) | 8 (1.9%) | 23 (3.56%) |  |  |  |  |  |
|  | **Total** | **1074 (100%)** | **428 (100%)** | **646 (100%)** |  |  |  |  |  |
| In general, would you say your healthcare providers listen carefully to you? * | Always | 317 (29.5%) | 169 (39.5%) | 148 (22.91%) | 41.09 | 5 | 9.00×10⁻⁸ | 5.31×10⁻⁶ | 0.20 (0.14, 0.25) |
|  | I don't know. | 20 (1.9%) | 6 (1.4%) | 14 (2.17%) |  |  |  |  |  |
|  | Never | 28 (2.6%) | 10 (2.3%) | 18 (2.79%) |  |  |  |  |  |
|  | Often | 333 (31.0%) | 129 (30.1%) | 204 (31.58%) |  |  |  |  |  |
|  | Rarely | 76 (7.1%) | 28 (6.5%) | 48 (7.43%) |  |  |  |  |  |
|  | Sometimes | 300 (27.9%) | 86 (20.1%) | 214 (33.13%) |  |  |  |  |  |
|  | **Total** | **1074 (100%)** | **428 (100%)** | **646 (100%)** |  |  |  |  |  |
| In general, do your healthcare providers encourage you to bring someone with you to your appointments? * | Always | 109 (10.1%) | 25 (5.8%) | 84 (13.00%) | 563.92 | 5 | 1.26×10⁻¹¹⁹ | 7.43×10⁻¹¹⁸ | 0.72 (0.68, 0.77) |
|  | Often | 234 (21.8%) | 24 (5.6%) | 210 (32.51%) |  |  |  |  |  |
|  | Sometimes | 296 (27.6%) | 36 (8.4%) | 260 (40.25%) |  |  |  |  |  |
|  | Rarely | 101 (9.4%) | 38 (8.9%) | 63 (9.75%) |  |  |  |  |  |
|  | Never | 230 (21.4%) | 215 (50.2%) | 15 (2.32%) |  |  |  |  |  |
|  | Not Applicable / I don't know. | 104 (9.7%) | 90 (21.0%) | 14 (2.17%) |  |  |  |  |  |
|  | **Total** | **1074 (100%)** | **428 (100%)** | **646 (100%)** |  |  |  |  |  |
| In general, how confident are you that you know the things that you need to do to take care of and manage your health? * | Very confident | 311 (29.1%) | 200 (47.2%) | 111 (17.24%) | 115.74 | 4 | 4.34×10⁻²⁴ | 2.56×10⁻²² | 0.33 (0.27, 0.39) |
|  | Somewhat confident | 558 (52.2%) | 177 (41.7%) | 381 (59.16%) |  |  |  |  |  |
|  | Not very confident | 132 (12.4%) | 32 (7.5%) | 100 (15.53%) |  |  |  |  |  |
|  | Not at all confident | 52 (4.9%) | 13 (3.1%) | 39 (6.06%) |  |  |  |  |  |
|  | I don't know. | 15 (1.4%) | 2 (0.5%) | 13 (2.02%) |  |  |  |  |  |
|  | **Total** | **1068 (100%)** | **424 (100%)** | **644 (100%)** |  |  |  |  |  |
| In the last 12 months, was there ever a time when you received conflicting information about your healthcare from different healthcare providers such as your family doctor, specialists, or other healthcare providers including nurses, dieticians, staff at clinics, and so on? * | Yes | 490 (45.9%) | 140 (33.0%) | 350 (54.35%) | 51.49 | 2 | 6.61×10⁻¹² | 3.90×10⁻¹⁰ | 0.22 (0.16, 0.28) |
|  | No | 497 (46.5%) | 253 (59.7%) | 244 (37.89%) |  |  |  |  |  |
|  | I don't know. / I don't remember. | 81 (7.6%) | 31 (7.3%) | 50 (7.76%) |  |  |  |  |  |
|  | **Total** | **1068 (100%)** | **424 (100%)** | **644 (100%)** |  |  |  |  |  |
| Are you able to move around your home and neighbourhood without fear of falling or getting disoriented? | Yes | 859 (80.6%) | 358 (84.4%) | 501 (78.04%) | 12.12 | 2 | 2.33×10⁻³ | 0.138 | 0.11 (0.05, 0.17) |
|  | No | 172 (16.1%) | 61 (14.4%) | 111 (17.29%) |  |  |  |  |  |
|  | I don't know. | 35 (3.3%) | 5 (1.2%) | 30 (4.67%) |  |  |  |  |  |
|  | **Total** | **1066 (100%)** | **424 (100%)** | **642 (100%)** |  |  |  |  |  |
| In the last 12 months have you been to an emergency department (ED) because you were sick or for a health related problem? * | Yes | 533 (50.4%) | 166 (39.7%) | 367 (57.43%) | 118.04 | 2 | 2.34×10⁻²⁶ | 1.38×10⁻²⁴ | 0.33 (0.28, 0.39) |
|  | No | 431 (40.8%) | 247 (59.1%) | 184 (28.79%) |  |  |  |  |  |
|  | I don't know. / I don't remember. | 93 (8.8%) | 5 (1.2%) | 88 (13.77%) |  |  |  |  |  |
|  | **Total** | **1057 (100%)** | **418 (100%)** | **639 (100%)** |  |  |  |  |  |
| Did you have to return to the ED for the same reason that you either visited an ed or were hospitalized previously for? | Yes | 216 (40.5%) | 67 (40.4%) | 149 (40.60%) | 5.27 | 2 | .072 | 1.00 | 0.10 (0.01, 0.18) |
|  | No | 282 (52.9%) | 94 (56.6%) | 188 (51.23%) |  |  |  |  |  |
|  | I don't know. / I don't remember. | 35 (6.6%) | 5 (3.0%) | 30 (8.17%) |  |  |  |  |  |
|  | **Total** | **533 (100%)** | **166 (100%)** | **367 (100%)** |  |  |  |  |  |
| The last time you went to the ED, was it for a condition that you think could have been treated by your regular healthcare provider or other healthcare professional if he/she had been available? * | Yes | 343 (64.4%) | 80 (48.2%) | 263 (71.66%) | 29.64 | 2 | 3.66×10⁻⁷ | 2.16×10⁻⁵ | 0.24 (0.15, 0.32) |
|  | No | 153 (28.7%) | 73 (44.0%) | 80 (21.80%) |  |  |  |  |  |
|  | I don't know. / I don't remember. | 37 (6.9%) | 13 (7.8%) | 24 (6.54%) |  |  |  |  |  |
|  | **Total** | **533 (100%)** | **166 (100%)** | **367 (100%)** |  |  |  |  |  |
| The last time you went to the ED, which of the following was the main reason you went to the ED rather than to your regular healthcare provider or another healthcare professional? * | It was an emergency | 180 (33.8%) | 72 (43.4%) | 108 (29.43%) | 58.49 | 8 | 1.00×10⁻⁵ | 5.90×10⁻⁴ | 0.33 (0.25, 0.41) |
|  | My provider was not available. | 76 (14.3%) | 25 (15.1%) | 51 (13.90%) |  |  |  |  |  |
|  | I could not get an appointment with my provider. | 76 (14.3%) | 16 (9.6%) | 60 (16.35%) |  |  |  |  |  |
|  | It was faster to go to the emergency. | 87 (16.3%) | 13 (7.8%) | 74 (20.16%) |  |  |  |  |  |
|  | The ED was closer. | 40 (7.5%) | 5 (3.0%) | 35 (9.54%) |  |  |  |  |  |
|  | My provider advised me to go to the ED. | 37 (6.9%) | 18 (10.8%) | 19 (5.18%) |  |  |  |  |  |
|  | My regular healthcare provider works out of ED. | 20 (3.8%) | 3 (1.8%) | 17 (4.63%) |  |  |  |  |  |
|  | Other (Please specify.) | 13 (2.4%) | 12 (7.2%) | 1 (0.27%) |  |  |  |  |  |
|  | I don't know. / I don't remember. | 4 (0.8%) | 2 (1.2%) | 2 (0.54%) |  |  |  |  |  |
|  | **Total** | **533 (100%)** | **166 (100%)** | **367 (100%)** |  |  |  |  |  |
| The last time you went to the ED, when you left how confident were you that you had the information you needed to care for and manage the health problem for which you went to the ED? * | Very confident | 158 (29.6%) | 44 (26.5%) | 114 (31.06%) | 20.34 | 4 | 3.90×10⁻⁴ | 0.023 | 0.20 (0.11, 0.28) |
|  | Somewhat confident | 240 (45.0%) | 66 (39.8%) | 174 (47.41%) |  |  |  |  |  |
|  | Not very confident | 86 (16.1%) | 27 (16.3%) | 59 (16.08%) |  |  |  |  |  |
|  | Not at all confident | 46 (8.6%) | 27 (16.3%) | 19 (5.18%) |  |  |  |  |  |
|  | I don't know. / I don't remember. | 3 (0.6%) | 2 (1.2%) | 1 (0.27%) |  |  |  |  |  |
|  | **Total** | **533 (100%)** | **166 (100%)** | **367 (100%)** |  |  |  |  |  |
| In the last 12 months have you been hospitalized overnight? * | Yes | 326 (30.8%) | 63 (15.1%) | 263 (41.16%) | 125.73 | 2 | 4.98×10⁻²⁸ | 2.94×10⁻²⁶ | 0.34 (0.29, 0.4) |
|  | No | 677 (64.0%) | 352 (84.2%) | 325 (50.86%) |  |  |  |  |  |
|  | I don't know. / I don't remember. | 54 (5.1%) | 3 (0.7%) | 51 (7.98%) |  |  |  |  |  |
|  | **Total** | **1057 (100%)** | **418 (100%)** | **639 (100%)** |  |  |  |  |  |
| When you left the hospital were you provided with easy to follow instructions on whom to contact if you had a question about your treatment or if your condition became worse? | Yes | 195 (59.8%) | 38 (60.3%) | 157 (59.70%) | 0.04 | 2 | .979 | 1.00 | 0.01 (0.0, 0.12) |
|  | No | 103 (31.6%) | 20 (31.7%) | 83 (31.56%) |  |  |  |  |  |
|  | I don't know. / I don't remember. | 28 (8.6%) | 5 (7.9%) | 23 (8.75%) |  |  |  |  |  |
|  | **Total** | **326 (100%)** | **63 (100%)** | **263 (100%)** |  |  |  |  |  |
| After you were discharged from hospital did your regular healthcare provider or other healthcare professional seem informed and up to date about the care you received in the hospital? | Yes | 189 (58.0%) | 31 (49.2%) | 158 (60.08%) | 3.33 | 3 | .341 | 1.00 | 0.10 (0.0, 0.21) |
|  | No | 72 (22.1%) | 15 (23.8%) | 57 (21.67%) |  |  |  |  |  |
|  | I have not seen my regular provider or other healthcare professionals since being discharged from hospital. | 47 (14.4%) | 13 (20.6%) | 34 (12.93%) |  |  |  |  |  |
|  | I don't know. / I don't remember. | 18 (5.5%) | 4 (6.3%) | 14 (5.32%) |  |  |  |  |  |
|  | **Total** | **326 (100%)** | **63 (100%)** | **263 (100%)** |  |  |  |  |  |
| In the last 12 months have you seen a medical specialist? This includes an appointment in person, by phone, video, email, or secure message.* | Yes | 615 (58.2%) | 248 (59.3%) | 367 (57.43%) | 29.13 | 2 | 4.72×10⁻⁷ | 2.79×10⁻⁵ | 0.17 (0.11, 0.23) |
|  | No | 375 (35.5%) | 164 (39.2%) | 211 (33.02%) |  |  |  |  |  |
|  | I don't know. / I don't remember. | 67 (6.3%) | 6 (1.4%) | 61 (9.55%) |  |  |  |  |  |
|  | **Total** | **1057 (100%)** | **418 (100%)** | **639 (100%)** |  |  |  |  |  |
| How would you rate the length of time it took between making the appointment and the actual visit?* | About right | 192 (31.1%) | 110 (44.0%) | 82 (22.34%) | 47.04 | 3 | 1.00×10⁻⁵ | 5.90×10⁻⁴ | 0.28 (0.2, 0.35) |
|  | Somewhat too long | 291 (47.2%) | 78 (31.2%) | 213 (58.04%) |  |  |  |  |  |
|  | Much too long | 122 (19.8%) | 56 (22.4%) | 66 (17.98%) |  |  |  |  |  |
|  | I don't know. / I don't remember. | 12 (1.9%) | 6 (2.4%) | 6 (1.63%) |  |  |  |  |  |
|  | **Total** | **617 (100%)** | **250 (100%)** | **367 (100%)** |  |  |  |  |  |
| When you last saw the specialist did he/she have basic medical information from your regular healthcare provider about the reason for your visit? | Yes | 451 (73.5%) | 164 (66.4%) | 287 (78.20%) | 13.74 | 2 | 1.04×10⁻³ | 0.061 | 0.15 (0.07, 0.23) |
|  | No | 116 (18.9%) | 54 (21.9%) | 62 (16.89%) |  |  |  |  |  |
|  | I don't know. / I don't remember. | 47 (7.7%) | 29 (11.7%) | 18 (4.90%) |  |  |  |  |  |
|  | **Total** | **614 (100%)** | **247 (100%)** | **367 (100%)** |  |  |  |  |  |
| After you saw the specialist did your regular healthcare provider seem informed and up to date about the care you got from the specialist?* | Yes | 384 (62.4%) | 120 (48.4%) | 264 (71.93%) | 48.97 | 2 | 2.32×10⁻¹¹ | 1.37×10⁻⁹ | 0.28 (0.21, 0.36) |
|  | No | 152 (24.7%) | 71 (28.6%) | 81 (22.07%) |  |  |  |  |  |
|  | I don't know. / I don't remember. | 79 (12.8%) | 57 (23.0%) | 22 (5.99%) |  |  |  |  |  |
|  | **Total** | **615 (100%)** | **248 (100%)** | **367 (100%)** |  |  |  |  |  |
| Has your health gotten worse because you were not able to access a specialist?* | Yes | 109 (29.1%) | 32 (19.5%) | 77 (36.49%) | 15.36 | 2 | 4.61×10⁻⁴ | 0.027 | 0.20 (0.10, 0.30) |
|  | No | 187 (49.9%) | 87 (53.0%) | 100 (47.39%) |  |  |  |  |  |
|  | Not Applicable / I have not had the need to see a specialist. | 79 (21.1%) | 45 (27.4%) | 34 (16.11%) |  |  |  |  |  |
|  | **Total** | **375 (100%)** | **164 (100%)** | **211 (100%)** |  |  |  |  |  |
| In the last 12 months when receiving care for a medical problem, was there ever a time when test results were not available at the time of a scheduled appointment with your provider?* | Yes | 339 (32.1%) | 80 (19.1%) | 259 (40.53%) | 54.72 | 3 | 7.89×10⁻¹² | 4.66×10⁻¹⁰ | 0.23 (0.17, 0.29) |
|  | No | 529 (50.0%) | 256 (61.2%) | 273 (42.72%) |  |  |  |  |  |
|  | I don't know. / I don't remember. | 57 (5.4%) | 26 (6.2%) | 31 (4.85%) |  |  |  |  |  |
|  | Not Applicable / I did not have any tests in the last 12 months. | 132 (12.5%) | 56 (13.4%) | 76 (11.89%) |  |  |  |  |  |
|  | **Total** | **1057 (100%)** | **418 (100%)** | **639 (100%)** |  |  |  |  |  |
| In the last 12 months have you looked at your medical or health records using online portals or digital tools that are designed for people with specific health conditions?* | Yes | 651 (61.9%) | 147 (35.6%) | 504 (79.00%) | 326.57 | 2 | 1.22×10⁻⁷¹ | 7.19×10⁻⁷⁰ | 0.56 (0.51, 0.61) |
|  | No | 350 (33.3%) | 256 (62.0%) | 94 (14.73%) |  |  |  |  |  |
|  | I don't know. / I don't remember. | 50 (4.8%) | 10 (2.4%) | 40 (6.27%) |  |  |  |  |  |
|  | **Total** | **1051 (100%)** | **413 (100%)** | **638 (100%)** |  |  |  |  |  |
| Which of the following is the main reason you have not looked at your medical records online?* | I did not want to check my medical records this way. | 27 (7.7%) | 20 (7.8%) | 7 (7.45%) | 43.78 | 7 | 1.00×10⁻⁵ | 5.90×10⁻⁴ | 0.35 (0.26, 0.45) |
|  | My provider does not make medical records available this way. | 51 (14.6%) | 34 (13.3%) | 17 (18.09%) |  |  |  |  |  |
|  | I do not know how to. | 76 (21.7%) | 57 (22.3%) | 19 (20.21%) |  |  |  |  |  |
|  | I do not have reliable/any access to the internet. | 41 (11.7%) | 18 (7.0%) | 23 (24.47%) |  |  |  |  |  |
|  | I do not have reliable/any access to tools needed (including computer, laptop, tablet, etc.) | 22 (6.3%) | 10 (3.9%) | 12 (12.77%) |  |  |  |  |  |
|  | I had no need to look at my medical records. | 61 (17.4%) | 51 (19.9%) | 10 (10.64%) |  |  |  |  |  |
|  | I never knew you could do this. | 55 (15.7%) | 51 (19.9%) | 4 (4.26%) |  |  |  |  |  |
|  | I don't know. | 17 (4.9%) | 15 (5.9%) | 2 (2.13%) |  |  |  |  |  |
|  | **Total** | **350 (100%)** | **256 (100%)** | **94 (100%)** |  |  |  |  |  |
| In the last 12 months have you looked at your medical records using an online portal or digital tool? This does not include being able to access results of lab tests completed at labs such as Lifelabs or Dynacare and provided by the lab.* | Yes | 460 (43.8%) | 53 (12.8%) | 407 (63.79%) | 252.14 | 2 | 1.77×10⁻⁵⁵ | 1.04×10⁻⁵³ | 0.49 (0.44, 0.54) |
|  | No | 522 (49.7%) | 348 (84.3%) | 174 (27.27%) |  |  |  |  |  |
|  | I don't know. / I don't remember. | 69 (6.6%) | 12 (2.9%) | 57 (8.93%) |  |  |  |  |  |
|  | **Total** | **1051 (100%)** | **413 (100%)** | **638 (100%)** |  |  |  |  |  |
| Other than for booking an appointment, in the last 12 months, have you used any of the following types of virtual methods to communicate with your regular healthcare provider or other healthcare professional about your medical care?* | Telephone | 204 (17.7%) | 159 (32.7%) | 45 (6.74%) | 457.09 | 9 | 1.00×10⁻⁵ | 5.90×10⁻⁴ | 0.63 (0.58, 0.67) |
|  | Email | 138 (12.0%) | 12 (2.5%) | 126 (18.86%) |  |  |  |  |  |
|  | Website | 65 (5.6%) | 9 (1.9%) | 56 (8.38%) |  |  |  |  |  |
|  | Video call | 37 (3.2%) | 9 (1.9%) | 28 (4.19%) |  |  |  |  |  |
|  | Texting | 35 (3.0%) | 4 (0.8%) | 31 (4.64%) |  |  |  |  |  |
|  | Multiple | 458 (39.7%) | 108 (22.2%) | 350 (52.40%) |  |  |  |  |  |
|  | Other | 2 (0.2%) | 2 (0.4%) | 0 (0.00%) |  |  |  |  |  |
|  | Did not communicate | 103 (8.9%) | 101 (20.8%) | 2 (0.30%) |  |  |  |  |  |
|  | I don't know | 9 (0.8%) | 9 (1.9%) | 0 (0.00%) |  |  |  |  |  |
|  | Missing | 103 (8.9%) | 73 (15.0%) | 30 (4.49%) |  |  |  |  |  |
|  | **Total** | **1154 (100%)** | **486 (100%)** | **668 (100%)** |  |  |  |  |  |
| How old are you?* | Under 18 years old | 8 (0.8%) | 1 (0.2%) | 7 (1.11%) | 307.36 | 7 | 1.00×10⁻⁵ | 5.90×10⁻⁴ | 0.54 (0.49, 0.6) |
|  | 18-24 years old | 159 (15.4%) | 14 (3.4%) | 145 (23.09%) |  |  |  |  |  |
|  | 25-44 years old | 464 (44.8%) | 116 (28.5%) | 348 (55.41%) |  |  |  |  |  |
|  | 45-64 years old | 246 (23.8%) | 131 (32.2%) | 115 (18.31%) |  |  |  |  |  |
|  | 65-74 years old | 95 (9.2%) | 87 (21.4%) | 8 (1.27%) |  |  |  |  |  |
|  | 75-84 years old | 43 (4.2%) | 41 (10.1%) | 2 (0.32%) |  |  |  |  |  |
|  | 85 years old or older | 16 (1.5%) | 14 (3.4%) | 2 (0.32%) |  |  |  |  |  |
|  | Prefer not to answer | 4 (0.4%) | 3 (0.7%) | 1 (0.16%) |  |  |  |  |  |
|  | **Total** | **1035 (100%)** | **407 (100%)** | **628 (100%)** |  |  |  |  |  |
| Select the gender category you identify with.* | Woman | 546 (53.8%) | 299 (73.6%) | 247 (40.56%) | 138.28 | 6 | 1.00×10⁻⁵ | 5.90×10⁻⁴ | 0.37 (0.31, 0.43) |
|  | Man | 388 (38.2%) | 98 (24.1%) | 290 (47.62%) |  |  |  |  |  |
|  | Trans woman | 26 (2.6%) | 0 (0.0%) | 26 (4.27%) |  |  |  |  |  |
|  | Trans man | 33 (3.3%) | 1 (0.2%) | 32 (5.25%) |  |  |  |  |  |
|  | Two-Spirit | 15 (1.5%) | 1 (0.2%) | 14 (2.30%) |  |  |  |  |  |
|  | Another gender identity (Please specify.) | 2 (0.2%) | 2 (0.5%) | 0 (0.00%) |  |  |  |  |  |
|  | Prefer not to answer | 5 (0.5%) | 5 (1.2%) | 0 (0.00%) |  |  |  |  |  |
|  | **Total** | **1015 (100%)** | **406 (100%)** | **609 (100%)** |  |  |  |  |  |
| Select the sexual orientation you identify with.* | Bisexual | 130 (12.6%) | 17 (4.2%) | 113 (17.97%) | 143.40 | 6 | 1.00×10⁻⁵ | 5.90×10⁻⁴ | 0.37 (0.32, 0.43) |
|  | Heterosexual (Straight) | 719 (69.5%) | 336 (83.0%) | 383 (60.89%) |  |  |  |  |  |
|  | Homosexual (Gay/Lesbian) | 83 (8.0%) | 11 (2.7%) | 72 (11.45%) |  |  |  |  |  |
|  | Queer | 41 (4.0%) | 5 (1.2%) | 36 (5.72%) |  |  |  |  |  |
|  | Two-Spirit | 22 (2.1%) | 1 (0.2%) | 21 (3.34%) |  |  |  |  |  |
|  | Another sexual orientation (Please specify.) | 3 (0.3%) | 3 (0.7%) | 0 (0.00%) |  |  |  |  |  |
|  | Prefer not to answer | 36 (3.5%) | 32 (7.9%) | 4 (0.64%) |  |  |  |  |  |
|  | **Total** | **1034 (100%)** | **405 (100%)** | **629 (100%)** |  |  |  |  |  |
| Select the race(s)/ethnicity(ies) you identify with.* | White | 611 (59.0%) | 354 (86.8%) | 257 (40.92%) | 290.15 | 9 | 1.00×10⁻⁵ | 5.90×10⁻⁴ | 0.53 (0.48, 0.58) |
|  | Asian | 96 (9.3%) | 5 (1.2%) | 91 (14.49%) |  |  |  |  |  |
|  | Black | 102 (9.8%) | 7 (1.7%) | 95 (15.13%) |  |  |  |  |  |
|  | Caribbean | 23 (2.2%) | 0 (0.0%) | 23 (3.66%) |  |  |  |  |  |
|  | Indigenous | 75 (7.2%) | 7 (1.7%) | 68 (10.83%) |  |  |  |  |  |
|  | Latin | 37 (3.6%) | 0 (0.0%) | 37 (5.89%) |  |  |  |  |  |
|  | Middle Eastern | 21 (2.0%) | 0 (0.0%) | 21 (3.34%) |  |  |  |  |  |
|  | Multi Race | 53 (5.1%) | 17 (4.2%) | 36 (5.73%) |  |  |  |  |  |
|  | Option Not Listed | 3 (0.3%) | 3 (0.7%) | 0 (0.00%) |  |  |  |  |  |
|  | Prefer Not to Answer | 15 (1.4%) | 15 (3.7%) | 0 (0.00%) |  |  |  |  |  |
|  | **Total** | **1036 (100%)** | **408 (100%)** | **628 (100%)** |  |  |  |  |  |
| When you see or speak with nurses doctors physicians specialists and others in the healthcare system in what official language are you most comfortable? | English | 981 (95.2%) | 380 (93.4%) | 601 (96.31%) | 9.28 | 2 | .010 | .595 | 0.09 (0.03, 0.16) |
|  | French | 41 (4.0%) | 25 (6.1%) | 16 (2.56%) |  |  |  |  |  |
|  | I am most comfortable in another language. (Please specify.) | 9 (0.9%) | 2 (0.5%) | 7 (1.12%) |  |  |  |  |  |
|  | **Total** | **1031 (100%)** | **407 (100%)** | **624 (100%)** |  |  |  |  |  |
| Do you have difficulty paying all your bills at the end of the month?* | Always | 89 (8.7%) | 33 (8.1%) | 56 (9.05%) | 253.53 | 4 | 1.13×10⁻⁵³ | 6.66×10⁻⁵² | 0.50 (0.44, 0.55) |
|  | Never | 268 (26.1%) | 214 (52.6%) | 54 (8.72%) |  |  |  |  |  |
|  | Prefer not to answer | 30 (2.9%) | 11 (2.7%) | 19 (3.07%) |  |  |  |  |  |
|  | Rarely | 207 (20.2%) | 54 (13.3%) | 153 (24.72%) |  |  |  |  |  |
|  | Sometimes | 432 (42.1%) | 95 (23.3%) | 337 (54.44%) |  |  |  |  |  |
|  | **Total** | **1026 (100%)** | **407 (100%)** | **619 (100%)** |  |  |  |  |  |
| After paying your monthly bills do you typically have enough money left for food?* | Always | 379 (36.8%) | 287 (70.9%) | 92 (14.74%) | 347.61 | 4 | 5.75×10⁻⁷⁴ | 3.39×10⁻⁷² | 0.58 (0.53, 0.63) |
|  | Sometimes | 394 (38.3%) | 59 (14.6%) | 335 (53.69%) |  |  |  |  |  |
|  | Rarely | 185 (18.0%) | 32 (7.9%) | 153 (24.52%) |  |  |  |  |  |
|  | Never | 51 (5.0%) | 17 (4.2%) | 34 (5.45%) |  |  |  |  |  |
|  | Prefer not to answer | 20 (1.9%) | 10 (2.5%) | 10 (1.60%) |  |  |  |  |  |
|  | **Total** | **1029 (100%)** | **405 (100%)** | **624 (100%)** |  |  |  |  |  |
| Do you ever worry about losing your place to live?* | Always | 80 (7.8%) | 34 (8.4%) | 46 (7.38%) | 233.58 | 4 | 2.23×10⁻⁴⁹ | 1.32×10⁻⁴⁷ | 0.48 (0.42, 0.53) |
|  | Sometimes | 376 (36.5%) | 71 (17.4%) | 305 (48.96%) |  |  |  |  |  |
|  | Rarely | 213 (20.7%) | 52 (12.8%) | 161 (25.84%) |  |  |  |  |  |
|  | Never | 336 (32.6%) | 241 (59.2%) | 95 (15.25%) |  |  |  |  |  |
|  | Prefer not to answer | 25 (2.4%) | 9 (2.2%) | 16 (2.57%) |  |  |  |  |  |
|  | **Total** | **1030 (100%)** | **407 (100%)** | **623 (100%)** |  |  |  |  |  |
| How often do you feel isolated from others?* | Always | 90 (8.8%) | 33 (8.1%) | 57 (9.27%) | 59.18 | 4 | 4.31×10⁻¹² | 2.55×10⁻¹⁰ | 0.24 (0.18, 0.3) |
|  | Sometimes | 348 (34.0%) | 118 (28.9%) | 230 (37.40%) |  |  |  |  |  |
|  | Rarely | 288 (28.2%) | 91 (22.3%) | 197 (32.03%) |  |  |  |  |  |
|  | Never | 278 (27.2%) | 163 (40.0%) | 115 (18.70%) |  |  |  |  |  |
|  | Prefer not to answer | 19 (1.9%) | 3 (0.7%) | 16 (2.60%) |  |  |  |  |  |
|  | **Total** | **1023 (100%)** | **408 (100%)** | **615 (100%)** |  |  |  |  |  |
| How often do you feel left out?* | Always | 74 (7.2%) | 34 (8.4%) | 40 (6.43%) |  |  |  |  |  |
|  | Sometimes | 395 (38.4%) | 126 (31.0%) | 269 (43.25%) | 82.79 | 4 | 4.46×10⁻¹⁷ | 2.63×10⁻¹⁵ | 0.28 (0.23, 0.34) |
|  | Rarely | 321 (31.2%) | 97 (23.9%) | 224 (36.01%) |  |  |  |  |  |
|  | Never | 224 (21.8%) | 145 (35.7%) | 79 (12.70%) |  |  |  |  |  |
|  | Prefer not to answer | 14 (1.4%) | 4 (1.0%) | 10 (1.61%) |  |  |  |  |  |
|  | **Total** | **1028 (100%)** | **406 (100%)** | **622 (100%)** |  |  |  |  |  |
| How often do you feel that you lack companionship?* | Always | 86 (8.3%) | 38 (9.4%) | 48 (7.69%) | 151.17 | 4 | 1.14×10⁻³¹ | 6.74×10⁻³⁰ | 0.38 (0.33, 0.44) |
|  | Sometimes | 429 (41.7%) | 114 (28.1%) | 315 (50.48%) |  |  |  |  |  |
|  | Rarely | 253 (24.6%) | 75 (18.5%) | 178 (28.53%) |  |  |  |  |  |
|  | Never | 241 (23.4%) | 174 (42.9%) | 67 (10.74%) |  |  |  |  |  |
|  | Prefer not to answer | 21 (2.0%) | 5 (1.2%) | 16 (2.56%) |  |  |  |  |  |
|  | **Total** | **1030 (100%)** | **406 (100%)** | **624 (100%)** |  |  |  |  |  |
| What type of housing are you currently living in?* | Renting | 175 (16.9%) | 92 (22.5%) | 83 (13.28%) | 203.92 | 7 | 1.70×10⁻⁴⁰ | 1.00×10⁻³⁸ | 0.44 (0.39, 0.5) |
|  | Homeowner | 464 (44.9%) | 258 (63.2%) | 206 (32.96%) |  |  |  |  |  |
|  | Long-term care | 94 (9.1%) | 16 (3.9%) | 78 (12.48%) |  |  |  |  |  |
|  | Retirement home / Residence | 90 (8.7%) | 2 (0.5%) | 88 (14.08%) |  |  |  |  |  |
|  | Affordable Housing Programs (Subsidized housing, Rent-Geared-to-Income (RGI) housing, Rent supplement programs) | 166 (16.1%) | 22 (5.4%) | 144 (23.04%) |  |  |  |  |  |
|  | Domiciliary hospital | 16 (1.5%) | 0 (0.0%) | 16 (2.56%) |  |  |  |  |  |
|  | No current living arrangements (Person who is homeless, couch surfing, etc.) | 13 (1.3%) | 6 (1.5%) | 7 (1.12%) |  |  |  |  |  |
|  | Other (Please specify.) | 15 (1.5%) | 12 (2.9%) | 3 (0.48%) |  |  |  |  |  |
|  | **Total** | **1033 (100%)** | **408 (100%)** | **625 (100%)** |  |  |  |  |  |
| Who was were the main person or people that filled in this questionnaire?* | Me, the patient/client | 706 (68.3%) | 361 (88.5%) | 345 (55.20%) | 169.20 | 3 | 1.90×10⁻³⁶ | 1.12×10⁻³⁴ | 0.40 (0.35, 0.46) |
|  | A friend or relative of the patient/client | 172 (16.7%) | 8 (2.0%) | 164 (26.24%) |  |  |  |  |  |
|  | Both me, the patient/client, and a friend or relative, together | 98 (9.5%) | 9 (2.2%) | 89 (14.24%) |  |  |  |  |  |
|  | Me, the patient/client, with the help of a health professional | 57 (5.5%) | 30 (7.4%) | 27 (4.32%) |  |  |  |  |  |
|  | **Total** | **1033 (100%)** | **408 (100%)** | **625 (100%)** |  |  |  |  |  |

*significant after Bonferroni correction

Note: Statistical significance was assessed using a Bonferroni-adjusted significance threshold of p = .00085 applied to **uncorrected p-values**. Bonferroni-adjusted p-values are also reported for reference.
